# Hybrid de novo genome assembly and centromere characterization of the gray mouse lemur (*Microcebus murinus*)

**DOI:** 10.1186/s12915-017-0439-6

**Published:** 2017-11-16

**Authors:** Peter A. Larsen, R. Alan Harris, Yue Liu, Shwetha C. Murali, C. Ryan Campbell, Adam D. Brown, Beth A. Sullivan, Jennifer Shelton, Susan J. Brown, Muthuswamy Raveendran, Olga Dudchenko, Ido Machol, Neva C. Durand, Muhammad S. Shamim, Erez Lieberman Aiden, Donna M. Muzny, Richard A. Gibbs, Anne D. Yoder, Jeffrey Rogers, Kim C. Worley

**Affiliations:** 1 Department of Biology, Duke University, Durham, NC 27708 USA; 2 Human Genome Sequencing Center, Baylor College of Medicine, Houston, TX 77030 USA; 3 Department of Molecular and Human Genetics, Baylor College of Medicine, Houston, TX 77030 USA; 4 Department of Pharmacology and Cancer Biology, Duke University, Durham, NC 27710 USA; 5 Department of Molecular Genetics and Microbiology, Duke University, Durham, NC 27710 USA; 6 Kansas State University Bioinformatics Center, Division of Biology, Kansas State University, Manhattan, KS 66506 USA; 7 The Center for Theoretical Biological Physics, Rice University, Houston, TX 77005 USA; 8 Department of Computer Science, Rice University, Houston, TX 77005 USA; 9 Present address: Department of Genome Sciences, University of Washington, Seattle, WA 98195 USA; 10 Present address: Bristol Myers-Squibb, 420 W Round Grove Rd, Lewisville, TX 75067 USA; 11 Present address: New York Genome Center, 101 Avenue of the Americas, New York, NY 10013 USA

**Keywords:** Centromeres, de novo assembly, Hi-C, Optical maps, Physical maps, Super-scaffolding, Strepsirrhine primate

## Abstract

**Background:**

The de novo assembly of repeat-rich mammalian genomes using only high-throughput short read sequencing data typically results in highly fragmented genome assemblies that limit downstream applications. Here, we present an iterative approach to hybrid de novo genome assembly that incorporates datasets stemming from multiple genomic technologies and methods. We used this approach to improve the gray mouse lemur (*Microcebus murinus*) genome from early draft status to a near chromosome-scale assembly.

**Methods:**

We used a combination of advanced genomic technologies to iteratively resolve conflicts and super-scaffold the *M. murinus* genome.

**Results:**

We improved the *M. murinus* genome assembly to a scaffold N50 of 93.32 Mb. Whole genome alignments between our primary super-scaffolds and 23 human chromosomes revealed patterns that are congruent with historical comparative cytogenetic data, thus demonstrating the accuracy of our de novo scaffolding approach and allowing assignment of scaffolds to *M. murinus* chromosomes. Moreover, we utilized our independent datasets to discover and characterize sequences associated with centromeres across the mouse lemur genome. Quality assessment of the final assembly found 96% of mouse lemur canonical transcripts nearly complete, comparable to other published high-quality reference genome assemblies.

**Conclusions:**

We describe a new assembly of the gray mouse lemur (*Microcebus murinus*) genome with chromosome-scale scaffolds produced using a hybrid bioinformatic and sequencing approach. The approach is cost effective and produces superior results based on metrics of contiguity and completeness. Our results show that emerging genomic technologies can be used in combination to characterize centromeres of non-model species and to produce accurate de novo chromosome-scale genome assemblies of complex mammalian genomes.

**Electronic supplementary material:**

The online version of this article (doi:10.1186/s12915-017-0439-6) contains supplementary material, which is available to authorized users.

## Background

Genomic technologies have advanced rapidly over the past decade, allowing for many novel research opportunities for biologists examining the genetics of non-model species. Perhaps one of the most exciting areas of advancement has been in the field of genome sequencing and assembly, where it is now possible for individual researchers to produce genome assemblies for organisms of their choosing. However, despite these recent advancements, there remain significant challenges to the production of high-quality de novo eukaryotic genome assemblies. An ideal de novo whole genome assembly will be as continuous as possible (i.e., have minimal gaps), will accurately reflect the linear organization of chromosomes, and will contain few, if any, errors in nucleotide sequence. Such high-quality assemblies can be annotated with all the genomic features that biologists wish to investigate, including protein coding genes, non-coding genes, regulatory sequences, repetitive regions, and heterochromatic regions, including telomeres and centromeres. One fundamental challenge in the de novo assembly of complex eukaryotic genomes is the inability of many current DNA sequencing datatypes (and associated genome assembly algorithms) to completely resolve highly repetitive regions such as SINES, LINES, and heterochromatin (including centromeres) [1, 2]. The de novo assembly of repeat-rich genomes is especially problematic when using high-throughput short read technologies (often called next-generation sequencing or NGS). Methods that depend solely on traditional short read data typically result in fragmented and incomplete assemblies that impede many important areas of biological research (e.g., comparative genomics, gene discovery, genome evolution) [3–8]. Nevertheless, the low cost of NGS, combined with its success in producing high accuracy sequences, is driving the production of many new de novo genome assemblies using solely NGS data.

Addressing the current shortcomings of NGS-exclusive de novo mammalian genome assemblies, without incurring the cost of generating deep long-read data (e.g. the recent gorilla assembly; [9]), requires complementary methods that can greatly improve scaffold lengths and fill gaps within these scaffolds using relatively low-coverage, long-read sequence data [10–12]. With respect to the de novo assembly of primate genomes, including human, long-range genomic information must be used to resolve and span highly repetitive regions and generate chromosome-scale assemblies [7, 9, 11, 13]. Recent advances in single-molecule sequencing and NGS sequencing library construction now allow for the production of long-range genomic data in various forms. These long-range technologies and methods are powerful, rapidly improving and, at the time of this writing, include long-read single-molecule DNA sequence data (e.g., Pacific Biosciences (PacBio) *RSII* and Sequel, or Oxford Nanopore MinION and PromethION), physical maps of individual DNA molecules (e.g., BioNano Genomics Irys and OpGen Argus), genome-wide chromatin interaction data (e.g., Hi-C, Dovetail Genomics), and genome-wide barcoded and localized linked-reads (e.g., 10X Genomics). Hybrid de novo genome assembly approaches that utilize combinations of these diverse technologies alongside fragmented yet high-quality de novo NGS contigs have the potential not only to resolve and span structural variants and repetitive regions, but also to generate accurate chromosome-scale scaffolds [11].

Complementary orthogonal technologies, such as high-resolution, whole-genome physical maps, can be used to identify and correct genome assembly errors and can also be mined for complex or repetitive sequence patterns of biological significance. As an example, physical maps can characterize highly repetitive regions of the genome that span millions of bases in length but are otherwise notoriously difficult to sequence and assemble. These genomic features, such as heterochromatin, are largely absent from most genome assemblies, thus reducing their biological applicability. In the human reference assembly the tandemly organized alpha-satellite DNA associated with centromeres has proven nearly impossible to fully sequence and assemble using existing approaches [14–16]. Although centromeres play a fundamental role in eukaryotic cell division and are essential for chromosome stability, the field of centromere biology faces numerous challenges and a molecular characterization of centromeres from various research organisms is not yet available.

**Fig. 1:**
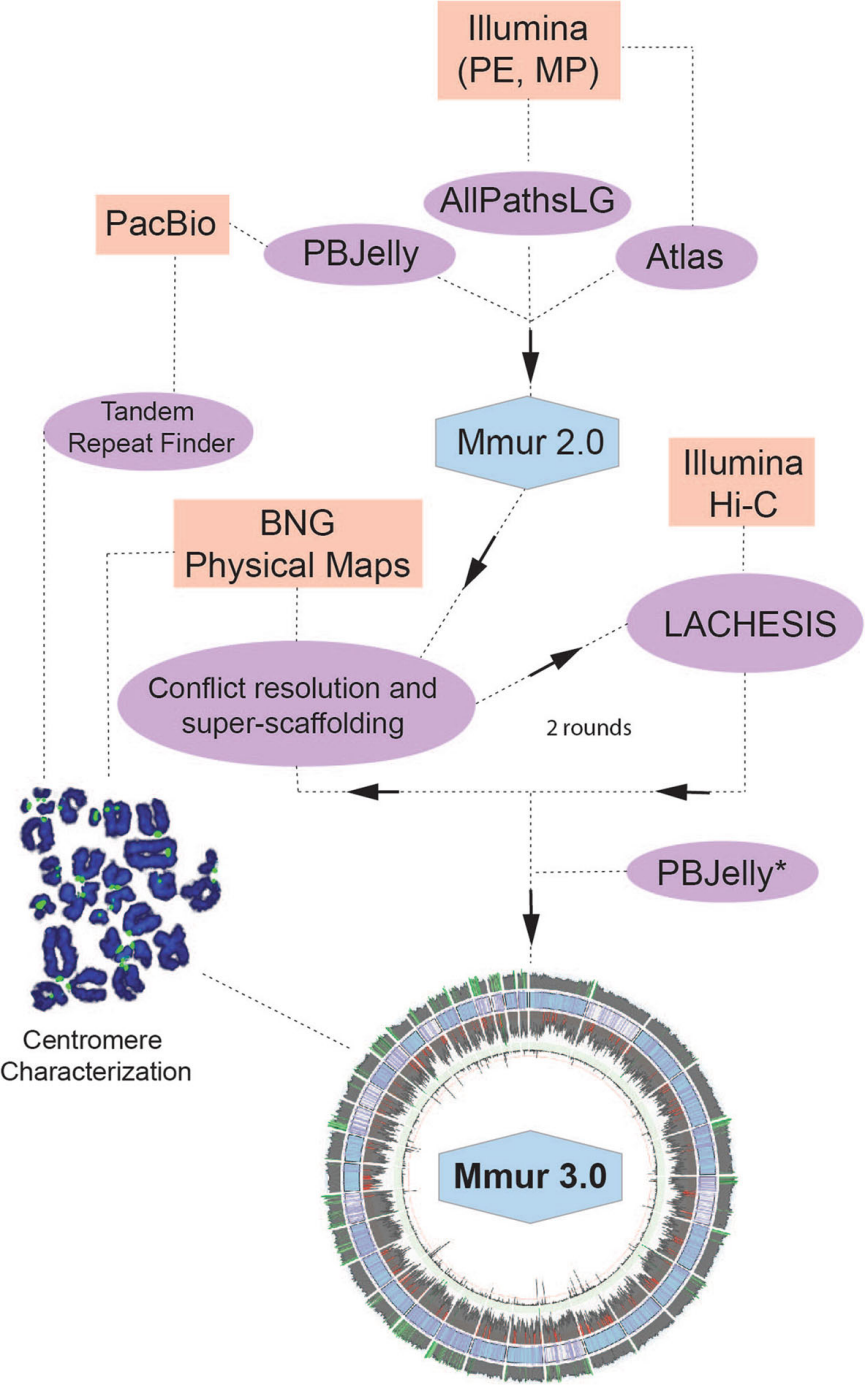
Flowchart of hybrid assembly procedure. The initial assembly was generated using Illumina data and AllPaths-LG, followed by refined scaffolding using Atlas-Link and gap filling using Atlas-GapFill. Further gap filling with PacBio data and PBJelly followed, generating Mmur 2.0. The Mmur 2.0 assembly was super-scaffolded in an iterative method using BNG optical map data to identify conflicts, break and join scaffolds, and Lachesis and Hi-C proximity ligation data to further super-scaffold. The PBJelly method was used a second time to fill gaps in the final super-scaffolds followed by Pilon error correction, creating the Mmur 3.0 assembly (* indicates that the same PacBio data was used for the second PBJelly analysis)

Here, we present an iterative approach to hybrid de novo genome assembly that incorporates datasets stemming from multiple genomic technologies and methods, namely Illumina, PacBio, Hi-C, and BioNano (Fig. 1, Additional file 1: Figure S1). We selected these particular technologies and library construction methods because they have been shown to produce high-quality, chromosome-scale assemblies when used together and are ideally suited for hybrid genome assembly of mammalian genomes [17]. We used a hybrid approach to improve the gray mouse lemur (*Microcebus murinus*; genome size ~2.7 Gb) genome from early draft status to a near chromosome-scale assembly, with contig and scaffold N50 values that are comparable to, or exceed, those of recently released non-human primate genomes [9, 18]. The gray mouse lemur is the only lemuriform primate known to routinely and spontaneously develop Alzheimer’s disease-like pathologies in captive populations and therefore is of intense interest for biomedical research focused on aging, translational disease research, and the convergent evolution of disease [19–22]. Moreover, as members of the strepsirrhine primate clade (Lemuriformes plus Lorisformes), mouse lemurs are representatives of the sister lineage to the haplorrhine primates (apes, including humans, Old World monkeys, New World monkeys, and tarsiers). Their position in the primate evolutionary tree makes mouse lemurs especially informative concerning the content and function of the ancestral (basal) primate genome. Thus, the availability of a robust high-quality annotated chromosome-scale assembly of the *M. murinus* genome will be beneficial to basic, comparative evolutionary, and translational research areas.

## Results

### Genome assembly, iterative conflict resolution, and Hi-C super-scaffolding

**Fig. 2:**
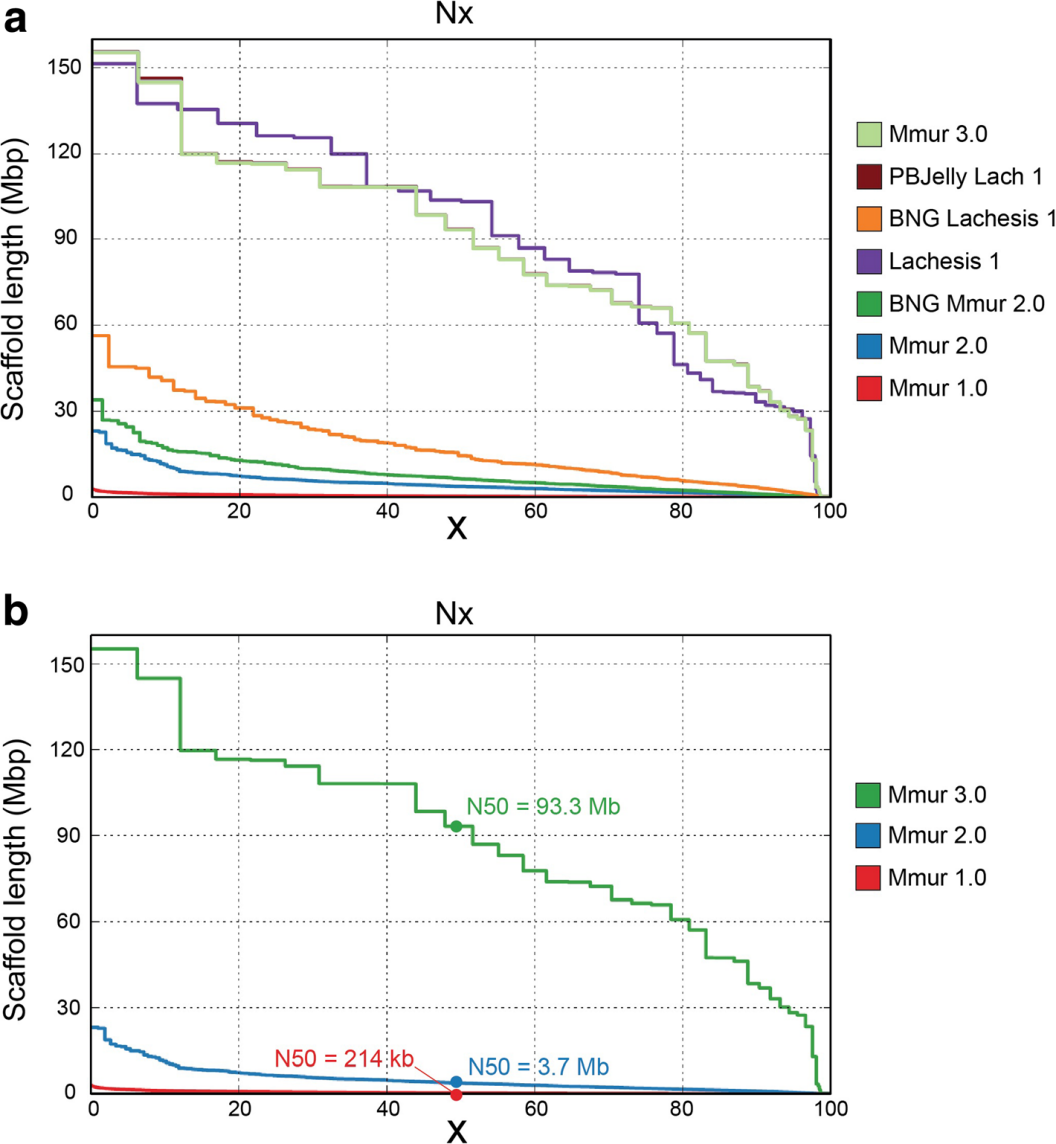
Hybrid iterative improvement of the mouse lemur genome assembly. **a** Graph showing improvement of the de novo mouse lemur genome assembly from draft status (Mmur 1.0) to chromosome-scale (Mmur 3.0) using the methods described herein. PBJelly Lach 1 results are coincident with those of Mmur 3.0. **b** Graph of the three main mouse lemur genome assemblies: Mmur 1.0 (draft assembly; ~1.93X) released in 2007; Mmur 2.0 (primary assembly; ~190X); Mmur 3.0 (final chromosome-scale assembly). For both panels, X-axis shows percent of genome contained within scaffolds (arranged according to length) and Y-axis shows scaffold length in Mb

In 2007, as part of the NHGRI Mammalian Genome Project, an initial draft low coverage assembly (1.93X Sanger sequencing) was released for the gray mouse lemur as Mmur 1.0 (contig N50 = 3.51 kb; scaffold N50 = 107.02 kb; Fig. 2; Table 1). Our primary genome assembly (Mmur 2.0) represents a major improvement, having a total sequence length of approximately 2.44 Gb, contig N50 of 182.9 kb and scaffold N50 of 3.7 Mb (longest scaffold 23 Mb; Fig. 2; Table 1; Additional file 2: Table S1). Our first super-scaffolding step identified and resolved 419 potential conflicts between in silico Mmur 2.0 restriction maps and consensus BNG physical maps (Additional file 2: Table S2), resulting in a scaffold N50 of 6.3 Mb with the longest super-scaffold being 33.9 Mb (Fig. 2, Table 1).

**Table 1:** Summary statistics for iterative super-scaffolding of the *Microcebus murinus* genome

	Mmur 1.0	Mmur 2.0	BNG Round 1	Lachesis Round 1	BNG Round 2	Lachesis Round 2	Mmur 3.0
Number of scaffolds	172,937	10,311	10,161	7813	8134	7679	7679
Total size of scaffolds, bp	2,910,103,014	2,438,804,424	2,469,090,855	2,492,570,855	2,491,435,191	2,495,985,191	2,487,714,386
Longest scaffold, bp	2,843,453	23,116,325	33,906,312	151,367,110	56,348,711	155,649,118	155,207,550
N50 scaffold length, bp	214,914	3,711,085	6,320,565	103,223.157	14,483,702	93,443,986	93,316,391
N50 contig length, bp	3511	182,929	182,011	182,011	181,924	181,924	234,304
Percentage of assembly in scaffolded contigs	95.4%	99.2%	99.2%	99.6%	99.6%	99.6%	99.6%
Scaffold, %N	36.35	2.5	3.7	4.6	4.56	4.74	4.07

For the second super-scaffolding step, Lachesis software (see Methods; [23]) clustered the majority of the assembly; specifically, 8470 contigs (83% of total contigs) representing 2.44 Gb (99%) of assembled sequence with 98% of the sequence within these clusters ordered (Additional file 2: Table S3). This increased scaffold N50 from 6.3 Mb to 103.22 Mb (Fig. 2, Table 1, Additional file 2: Table S1). A second iteration of these two super-scaffolding steps corrected 308 putative misjoins, clustered 6934 contigs (85% of total contigs) representing 2.47 Gb (99%) of assembled sequence, and ordered 98% of the total sequence length in these clusters. This increased scaffold N50 to 93.44 Mb (Fig. 2; Table 1; Tables S1 and S2) in the version that was subjected to the final gap-filling and error correction steps (below).

### Gap-filling and error correction

PBJelly [10] filled 4844 gaps in the improved scaffolds and extended sequence into additional gaps at one (4698) or both (1152) ends, resulting in 9,084,592 bp of additional sequence in the assembly (Additional file 2: Table S1). Sequence error polishing with Pilon [24] corrected 540,621 base substitutions, 791,550 insertions (totaling 1,032,222 bp), and 304,339 deletions (totaling 597,799 bp; Additional file 2: Table S4), resulting in the final Mmur 3.0 assembly. The larger number of corrected insertions compared to deletions is consistent with the PacBio error distribution of more insertions than deletions [25].

### Quality assessment of final chromosome-scale assembly (Mmur 3.0)

The final assembly had a length of 2.49 Gb (Table 1). This assembled genome size compares favorably to genome size estimates based on Illumina reads using PreQC [26] (2.44 Gb) and Jellyfish [27] (2.37 Gb), as well as estimates based on the Bionano map length (2.33 Gb).

**Fig. 3:**
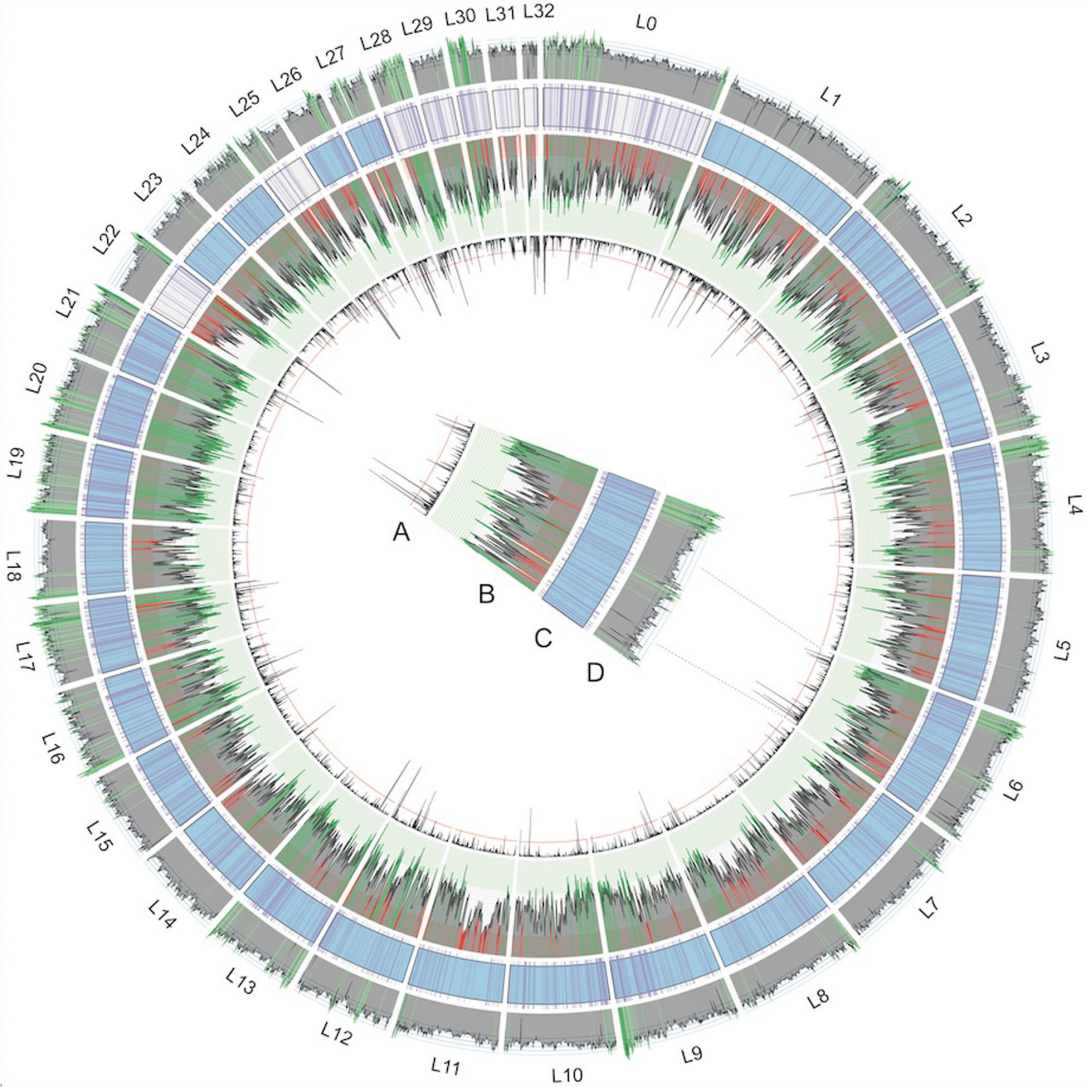
Mouse lemur 3.0 assembly. Circos plots were calculated with a sliding window of 500 kb. **a** Linear plot of percent of gaps encoded as N’s, plotted inward, where the red horizontal line is 25%. **b** Histogram of BNG physical map coverage across the scaffold, plotted with three horizontally shaded zones that match the data’s quartiles: 35× coverage and below is red (less than Q1), 35–56× coverage is grey (Q1–Q3), and 56× coverage and above is green (greater than Q3). **c** Lachesis scaffolds arranged according to length (in base pairs). Blue colored scaffolds represent those assigned to mouse lemur chromosomes (see Fig. 5) and white scaffolds are undetermined. Purple hashes identify regions containing the complete single copy genes (n = 2628) according to BUSCO analysis. **d** Histogram of percent of bases that are G + C across the genome. Genome-wide average is 40.98%, regions shaded light green are at least 47.5%, and regions shaded dark green are at least 55% G + C content

Ensembl mouse lemur canonical transcripts were mapped to the various assembly versions and the percent of the transcript length mapping was calculated (Additional file 2: Table S1). In the final version, 15,606 (96%) of protein coding transcripts were covered at 95–100% and 8448 (97%) of non-coding transcripts were full length. This was an increase relative to the Mmur 2.0 assembly of 121 (0.74%) protein coding transcripts and 50 (0.57%) non-coding transcripts. A stringent analysis of genes using BUSCO [28] identified 2700 genes that are present in full length, representing 89.32% of the 3023 genes in the BUSCO vertebrate dataset (Table 2; Fig. 3; Additional file 2: Table S1).

**Table 2:** Benchmarking Universal Single-Copy Orthologs (BUSCO) results based on 3023 groups searched

	Mmur 2.0	BNG Round 1	Lachesis Round 1	BNG Round 2	Lachesis Round 2	Mmur 3.0
Complete single-copy BUSCOs	2708	2686	2697	2706	2690	2700
Complete duplicated BUSCOs	75	74	68	73	65	72
Fragmented BUSCOs	188	206	183	189	189	191
Missing BUSCOs	127	131	143	128	144	132

Putative conflicts with the BNG map were reduced to 186 conflicts in the 291 scaffolds large enough to be evaluated by the BNG pipeline. Treating the remaining conflicts as gaps and examining the length distribution of conflict-free regions, 50% of the genome is held in 47 sequences (L50 = 47) and 75% of the genome in 102 sequences (L75 = 102), indicating expansive regions that are consistent with the physical maps (Additional file 3: Figure S2).

Sequence quality (base and indel error rates) was estimated using GATK to compare read data to Mmur 3.0. A caveat of these estimates is that the Illumina and PacBio data were from different mouse lemur individuals (see Methods), thus some homozygous alternative alleles may represent true biological differences. A total of 153,595 homozygous alternative SNPs were identified that may represent incorrect bases in the assembly, suggesting an estimated base error rate of 0.0064%. However, this estimate is an upper bound as polymorphism among the mouse lemur samples will account for some differences. There were 444,617 homozygous alternative indels, representing 830,957 bp differing from the assembly, which may represent small (<60 bp) local missassemblies or indel variants between the samples used to generate the sequence data (Additional file 2: Table S5).

### Whole genome alignment and assignment of mouse lemur chromosomes

**Fig. 4:**
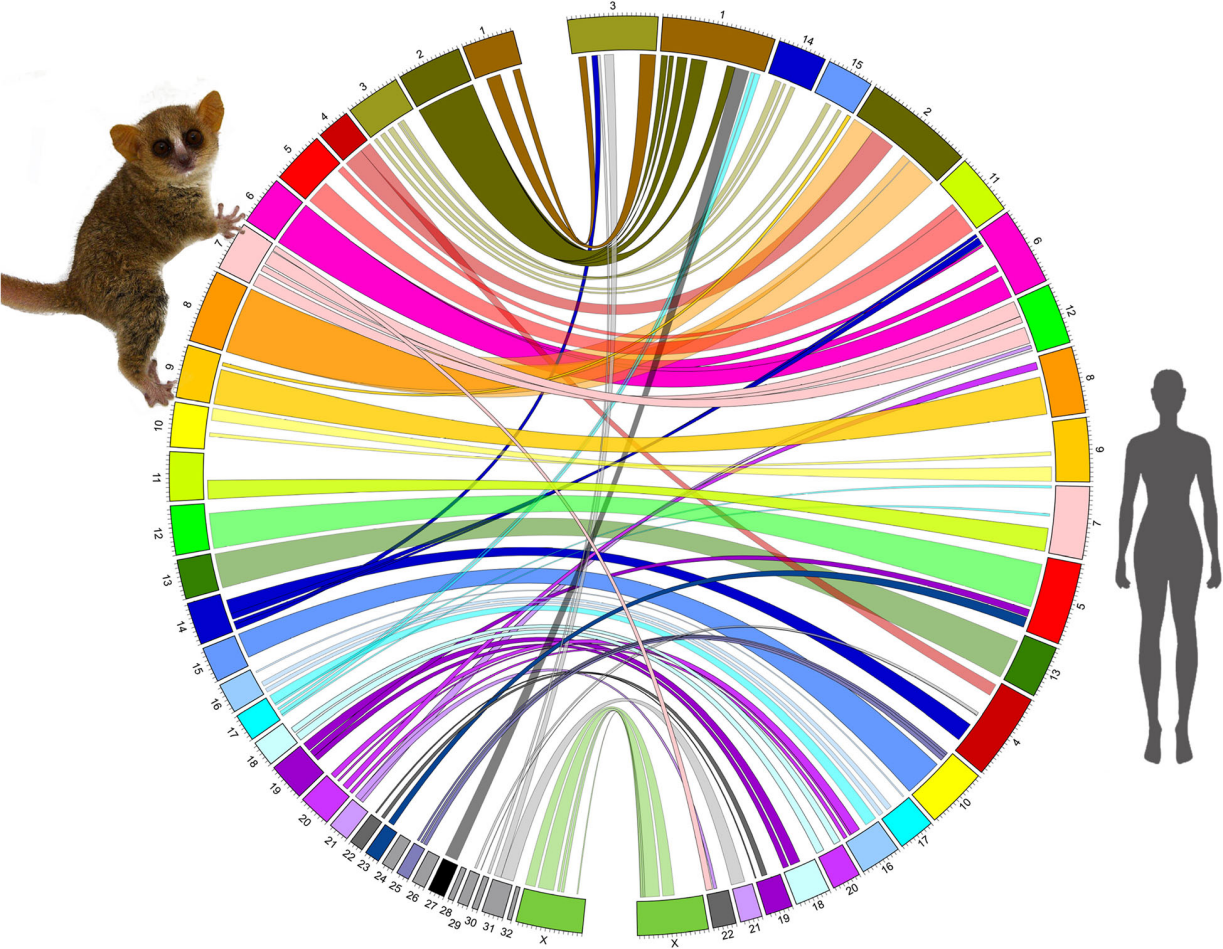
Macro synteny between mouse lemur and human chromosomes. Broad regions of synteny were identified between 33 *M. murinus* chromosomes (left) and 23 human chromosomes (right) using MUMmer software and these regions are shown using ribbons colored according to *M. murinus* chromosome number. Putative identifications for the 33 *M. murinus* chromosomes were based on comparative cytogenetic data [29] (see Results; Additional file 2: Tables S6 and S7; Additional file 4: Figure S3). Ticks in each chromosome indicate lengths of 10 Mb. Mouse lemur photo courtesy of David Haring and the Duke Lemur Center

Whole genome alignment between the 33 mouse lemur super-scaffolds and 23 human chromosomes revealed that major portions of each mouse lemur Lachesis group (ranging from 930 kb to 84.5 Mb) shared sequence homology with either one or two of the 22 human autosomes and X chromosomes (Additional file 4: Figure S3). In light of this result, we examined these alignments within the context of previously published comparative cytogenetic data [29]. These data show that seven *M. murinus* chromosomes have 1:1 relationships with specific human chromosomes, whereas seven have 2:1 relationships, two have 2:2 relationships, and one has 1:3 relationships (Additional file 2: Table S6). The alignment patterns observed between the 33 primary Lachesis scaffolds and the 23 human chromosomes were consistent with those observed using comparative cytogenetics. Using this information, we assigned 26 primary Lachesis groups to 23 mouse lemur chromosomes and we provided putative chromosome assignments to the remaining 10 mouse lemur chromosomes (Fig. 4; Additional file 2: Table S7 and Additional file 4: Figure S3); these served as the foundation for future FISH experiments to evaluate accuracy.

### Characterization of mouse lemur centromeres

**Fig. 5:**
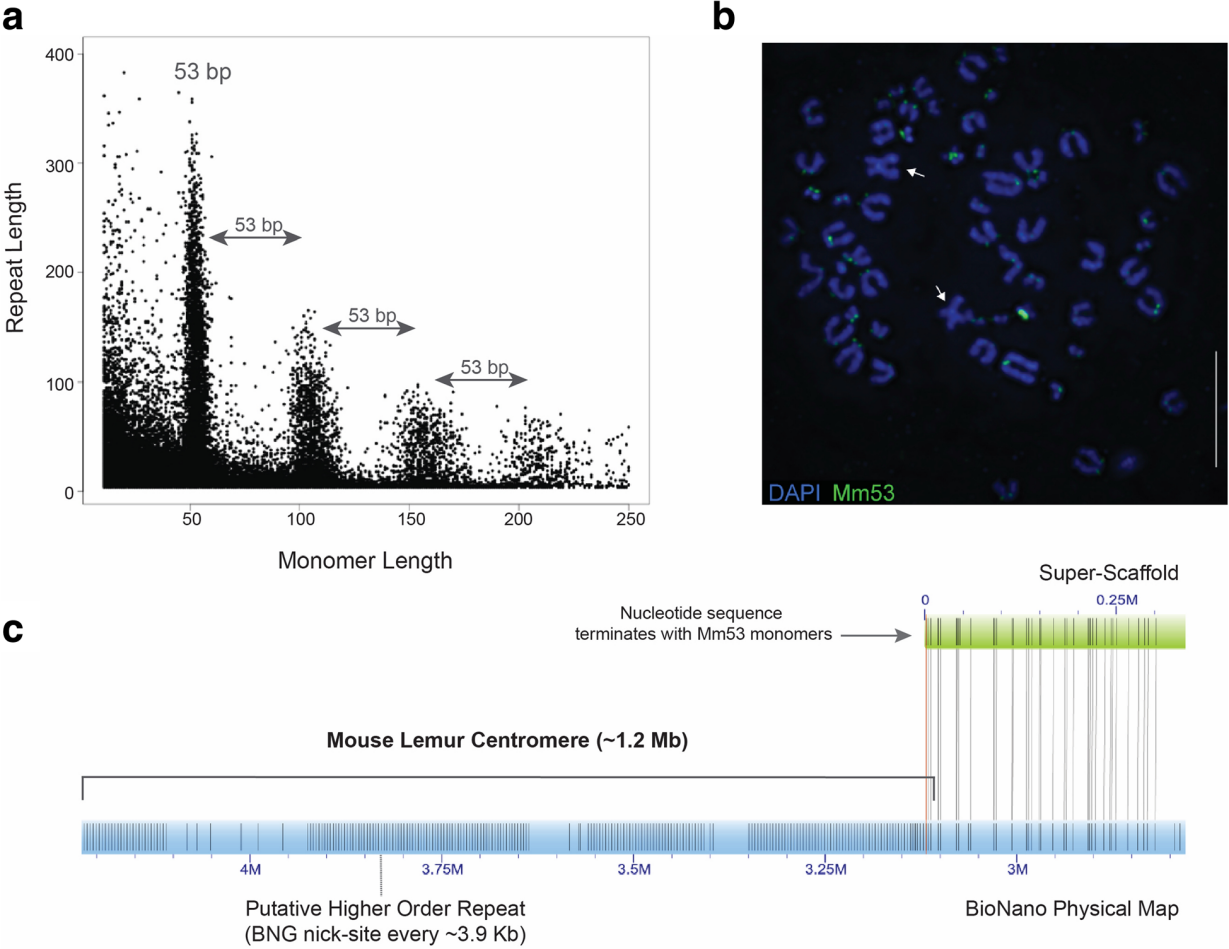
Centromere discovery using single molecule PacBio and BioNano data. **a** Graph of repeat units identified within raw PacBio data using Tandem Repeats Finder (see Methods). Each dot represents a repeat unit within a raw PacBio read and is graphed according to monomer length and overall (tandem) repeat length. A distinct distribution surrounding a 53 bp monomer was observed (including tandem repeats divisible by 53 bp). **b** The 53 bp monomer (Mm53) was identified, using FISH, to localize to nearly all centromeres in the mouse lemur karyotype, with the exception being the X chromosome (see Results and Fig. 6). **c** We mined our genome assembly for the Mm53 monomer associated with *M. murinus* centromeres. The Mm53 repeat was detected near the ends of scaffolds and/or gaps of Ns (representative in silico physical map shown in green). When aligned to consensus BioNano (BNG) physical map (blue), a distinct repeat unit was identified, indicating the presence of a BNG label within mouse lemur centromeres, thus providing a measure of higher-order repeat unit (~3.9 kb) and overall alpha-satellite array size within our BNG physical maps

**Fig. 6:**
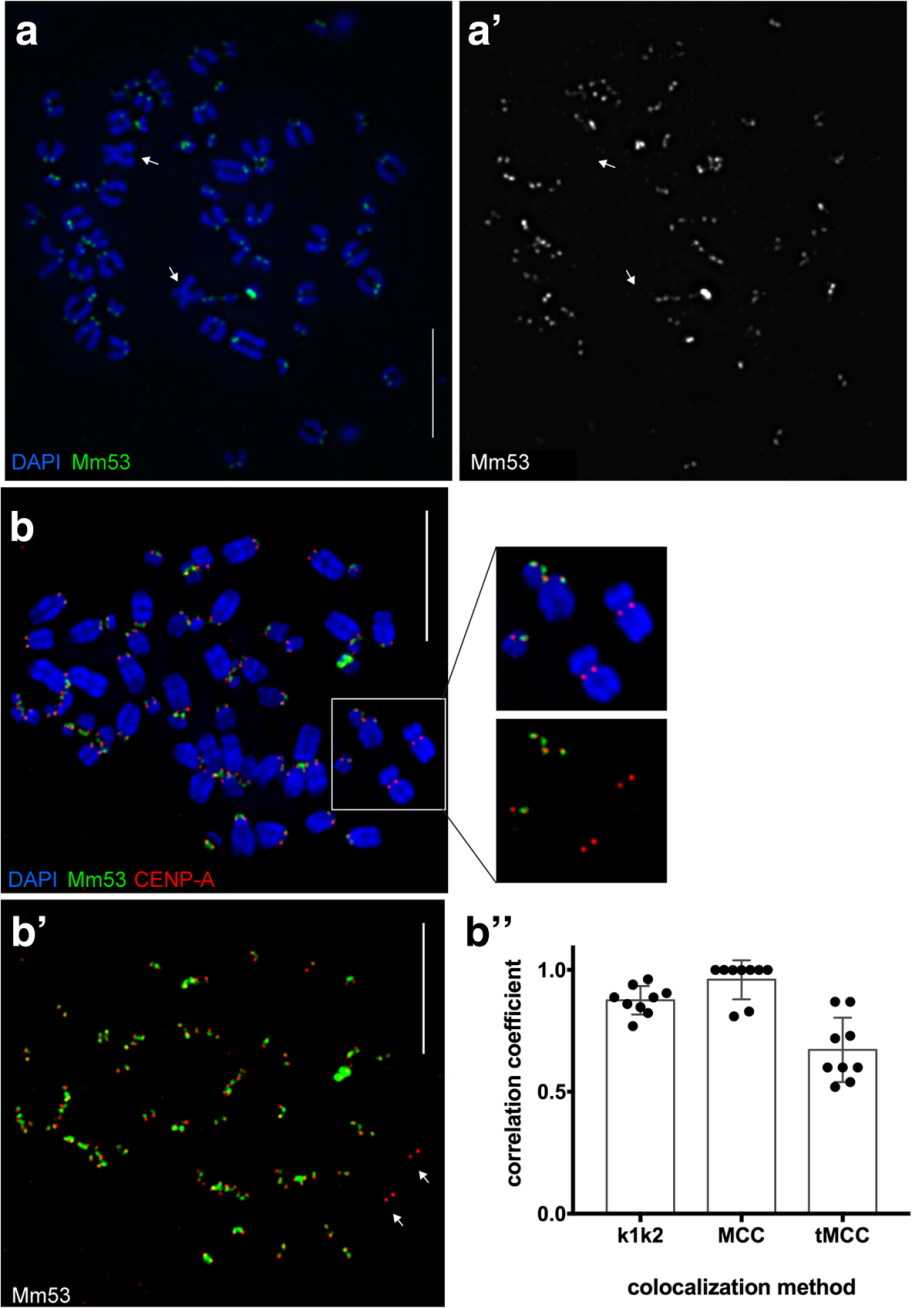
Functional identification of centromeric sequences in *M. murinus*. **a**, **a’**: Female mouse lemur metaphase chromosomes (blue) were hybridized with Mm53 (green), showing that the 53 bp sequence, Mm53, was present at every centromere except for the two metacentric X chromosomes (arrows). Gray-scale image shows the Mm53 fluorescent signal alone, illustrating the vast difference in abundance among the mouse lemur chromosomes. **b**–**b”**: Combined immunostaining for the essential centromere protein CENP-A and FISH with the Mm53 probe showed that CENP-A was present at every mouse lemur chromosome, including the two X chromosomes (insets in **b**). Gray scale images of fluorescent signals for Mm53 (**b’**) and CENP-A (**b”**) are separated out to emphasize relatively equal amounts of CENP-A at each chromosome, despite varying amounts of Mm53 centromeric sequence. The two X chromosomes have functional centromeres but lack Mm53, indicating that the X centromere is defined by a novel sequence that remains unidentified. Multiple colocalization analyses (k1k2 overlap coefficient and Manders’ colocalization coefficient (MCC), without and with thresholding) were performed on individual metaphases (n = 10 for each dot plot) to measure colocalization of red (CENP-A) and green (Mm53) signals. These analyses emphasized that a high proportion of CENP-A overlapped with Mm53

We identified 21,032 raw PacBio sequences containing repeat units meeting our Tandem Repeat Finder (TRF) threshold. Graphical output of the TRF PacBio results revealed a clear pattern centered around a 53 bp monomer: TCT-GCC-GTG-GGT-GAG-TGG-ACA-CAG-CCA-GAT-CCG-CAC-TGC-GCC-CTG-CCT-GCC-CG (Mm53; Fig. 5; Additional file 5: Figure S4). The genome-wide distribution of the Mm53 sequence, as revealed by fluorescence in situ hybridization (FISH), shows that the monomer appears at the primary constrictions of nearly every mouse lemur chromosome and is largely coincident with immunostaining for CENP-A, a protein component of mammalian centromeres (Fig. 6). The Mm53 monomer is not visible on the mouse lemur X chromosome (Fig. 6a, b), suggesting that the nucleotide composition of this centromere is defined by a different sequence motif.

A search of the final Mmur 3.0 assembly using TRF identified 1028 arrays greater than 2 kb (2002–75,974 bp). The Mm53 monomer was associated with 118 of these arrays (11.5%), varying in length between 2018 bp and 71,673 bp. Visual inspection of alignments between BNG physical maps and in silico Mmur 3.0 Mm53-containing scaffolds resulted in the identification of a highly repetitive BNG label pattern occurring within regions associated with the centromeric Mm53 monomer (Fig. 5; Additional file 6: Figure S5). The mean repeat unit for BNG regions associated with Mm53-containing Mmur 3.0 scaffolds was approximately 3.9 kb, a value comparable with higher-order repeats in primate centromeres [30]. We mined our raw BNG physical maps for additional regions containing repeat signatures and identified 35,079 raw BNG molecules containing 67,757 repeats varying in unit size (based on raw BNG molecules) from 2 kb to 35.5 kb. Repeat units of approximately 2.6 kb and 3.9 kb in length were enriched and the approximately 3.9 kb repeat unit was associated with the Mm53 monomer (Fig. 5), therefore indicating a higher-order array structure of mouse lemur centromeres (Additional file 7: Figure S6). We identified 29 consensus BNG physical maps containing putative higher-order repeat signatures, and these ranged in size from simple unordered arrays of approximately 400 kb to complex arrays spanning at least 3.2 Mb (Fig. 5; Additional file 8: Figure S7).

## Discussion

Employing an iterative analytical approach that integrates a diverse suite of sequencing, scaffolding, and physical mapping methods (Fig. 1), we produced a gray mouse lemur genome assembly (Mmur 3.0) that has long, high-quality contigs to support gene annotation and chromosome-scale scaffolds (Fig. 2). De novo assemblies for index species within clades of mammals for which no high-quality assembly is available, such as the strepsirrhine primates, open many new avenues for basic, evolutionary, and biomedical research [31]. The strepsirrhine primate clade contains more than 100 recognized living species, and is the sister clade to the haplorrhine primates, the group that includes all extant monkeys and apes, including humans. Our new assembly for the gray mouse lemur provides a high-quality reference that will be useful as a basis for comparative analyses of this species and all lemurs. This assembly also provides much improved resources for investigation of biomedical questions such as the basis of Alzheimer-related amyloid plaques and the origin of neurodegenerative disease and other pathologies of brain aging found to develop spontaneously in this species [19–22, 32].

### *M. murinus* 3.0 assembly quality

The mouse lemur assembly compares favorably to other recently produced non-human primate genome assemblies. Of the 27 non-human primate species with publicly available de novo genome assemblies, only the recent gorilla genome assembly (*Gorilla gorilla gorilla*; [9] has longer contigs, and only the vervet (*Chlorocebus aethiops sabaeus*) [18] has comparable scaffolding. The gorilla assembly, generated from 74.8X PacBio coverage of a reference animal and error corrected with a combined 194X Illumina coverage from seven individuals, has a reported contig N50 of 9.6 Mb. While the new gorilla contig N50 is substantially higher than our 234 kb contig N50, our scaffold N50 of 93.3 Mb is greater than the reported gorilla scaffold N50 of 23.1 Mb. The vervet genome was generated by merging a 100X Illumina assembly with a 19X 454 assembly, and employed a substantial amount of Sanger sequencing of BAC ends for scaffolding. That assembly has contig N50 of 90 kb and a scaffold N50 of 81 Mb [18], but these 454 and Sanger BES-based methods are not likely to be utilized for genomes in the future.

Different methods have been used to evaluate genomes [33, 34], but just as there is no single best assembly method (there are trade-offs between maximizing contiguity statistics vs. completeness vs. correctness), there is no single metric for determining the ‘best’ genome assembly. As genomes approach chromosome-scale scaffolds, comparing the scaffold N50 statistics between species becomes less informative since the upper limit to the scaffold size is the length of the chromosomes, and mammalian genomes are partitioned into varying numbers of chromosomes (between 6 and 102 diploid chromosomes) [35]. Comparing the fraction of the genome contained in the largest K scaffolds, where K is the number of chromosomes in the species, this mouse lemur Mmur 3.0 assembly and the vervet assembly [18] are the two best scaffolded non-human primate genomes available, containing 98% of the sequence within K scaffolds. The Mmur 3.0 assembly is also very complete as measured by alignments to transcripts and correct as measured by the more stringent BUSCO evaluations of correct orthologs.

### Utility of emerging genomic technologies for chromosome-scale mammalian genome assemblies

Although the mouse lemur assembly reported here satisfies many research needs for accurate contigs and scaffolds, it still falls short of the goal of a continuous gap-free sequence for each chromosome. Indeed, it is likely that our approach of producing an initial backbone assembly with the exclusive use of short-read whole-genome shotgun data fails to overcome the challenges associated with resolving highly repetitive regions, including large tandem and segmental duplications [8]. Moreover, additional work that focuses on improving the quality of the mouse lemur genome assembly must include the identification and correction of false duplications [36]. Future efforts to improve our assembly approach will be directed at obtaining more complete scaffolds, possibly through the utilization of accurate long-read, single-molecule sequencing data to produce the initial contigs and scaffolds followed by Hi-C chromatin-interaction data. This would help to resolve a greater percentage of bases within repetitive regions of initial contigs. It is also possible that cross-chromosome 3D interactions may be interfering with our ability to generate full chromosome length scaffolds. In light of this, it is likely that physical genome maps will remain an important independent tool for the accurate assembly of mammalian genomes, as such maps can be used to resolve inter-chromosome Hi-C assembly conflicts and to correct assembly errors associated with repeat-rich regions of the genome.

At the time of this writing, several substantial upgrades to long-read single-molecule sequencing (e.g., PacBio Sequel; Oxford Nanopore MinION) and mapping (e.g., BioNano Saphyr) technologies have been released, and it is likely that this sector of genomic technology will continue to be improved. Considering this, it is important to note that the hybrid genome assembly methods presented herein can be used interchangeably in a variety of ways to leverage the power of individual technological advancements. Our approach produced a high-quality reference mammalian genome that leveraged less expensive optical mapping and Hi-C data with lower-coverage PacBio data. Although physical (i.e., optical) mapping methods have been available for many years [37], the computation methods required to use these methods effectively for de novo genome applications were not widely available. The current BNG methods are finally benefiting from the useful available software needed to make these methods a common part of a genome assembly strategy. Hi-C methods are changing rapidly, although much of the focus of the improvements is on the analysis of 3D interactions; however, the use of 2D data for assembly scaffolding is also being addressed [38]. Nevertheless, combining the aggressive scaffolding provided by current Hi-C scaffolding methods (e.g., Lachesis [23]) with the orthogonal error correction and scaffolding that the BNG data provides, produces a highly contiguous, high-quality scaffolding that complements long contig assemblies.

### *M. murinus* centromere characterization

We provide the first characterization of specific repetitive sequences within the centromeres of a strepsirrhine primate (Figs. 5 and 6; Additional file 4: Figures S3, Additional file 5: Figures S4, Additional file 6: Figures S5, Additional file 7: Figures S6, and Additional file 8: Figures S7). This result underscores the power of independent genomic datasets to identify key genomic features that have previously been difficult to characterize. The methodologies underlying the discovery and description of the Mm53 monomer creates new opportunities for comparative analysis of centromeres across highly divergent primate lineages. Primate centromeres typically consist of long arrays of tandem repeats spanning millions of bases [14, 39, 40], which are woefully underrepresented in current genome assemblies. However, the repetitive nature of primate centromeres facilitates their discovery using a particular combination of data structure and bioinformatic strategy [41, 42]. In this study, we used a straightforward approach to identify and extract a 53 bp monomer (Mm53) associated with nearly all mouse lemur centromeres (Figs. 5 and 6), and then confirmed that this 53 bp monomer was enriched at primary constrictions and was coincident with centromere proteins. Moreover, our single-molecule BNG data allowed us to locate a potential higher-order repeating structure within the physical maps (Fig. 5; Additional file 6: Figures S5 and Additional file 8: Figure S7).

We recognize that the presence of a BNG nick-site within the mouse lemur centromere was a fortunate result that is unlikely to be replicated using non-targeted approaches in other non-model species. However, considering our results, it is possible for future projects to use our discovery method to mine long-read single-molecule sequence data for repeat signatures consistent with putative centromere higher-order repeats, and then use those results to select a suitable restriction enzyme for physical map characterization. This ‘reverse engineering’ approach could provide targeted physical maps spanning several megabases of centromere regions and would greatly advance the centromere biology of non-model organisms. The absence of the mouse lemur Mm53 monomer from the X chromosome indicates a pattern of inter-chromosomal difference that may be relevant to understanding sex chromosome identity and function (Fig. 6). This apparent divergence of mouse lemur X chromosome centromeres may have implications for both centromere function, sex chromosome evolution, and speciation within *M. murinus*.

### Taxonomic status of captive research colonies

*M. murinus* is endemic to the island of Madagascar, as are all species within the genus, and is part of an evolutionary radiation that has experienced explosive diversification during the last few million years [43, 44]. The *Microcebus* species are morphologically highly similar and therefore difficult to differentiate using traditional external phenotypic characters. Recognized as only two species in 1992 – *M. murinus* from western Madagascar and *M. rufus* from the eastern regions of the island – current taxonomy for the genus contains 25 named species, with the potential for additional species recognition with increased geographic sampling and consequent genetic characterization. To maximize the genetic diversity in the captive breeding colonies of *M. murinus* that were established in the 1960s and 1970s, individuals were intentionally sampled from across what was historically understood to represent the expansive geographic distribution of *M. murinus* in Madagascar. Therefore, genetic variation observed in captive research colonies may be inflated relative to the genetic variation observed in natural, independently evolving populations. Indeed, one of the most recent species to be recognized within the genus, *M. ganzhorni* [45], was until very recently considered to be a population within *M. murinus*. The new species designation of *M. ganzhorni* was justified on both genetic and biogeographic grounds, though confidence in species identity would be greatly enhanced with additional genomic information, and detailed morphological and ecological analysis. Until such time that these additional analyses can be performed, however, *M. ganzhorni* is perhaps best thought of as an independently evolving population lineage within the *M. murinus* complex [45].

During the course of our work on the Mmur 3.0 genome assembly we identified mtDNA cytochrome-*b* gene haplotypes of both *M. murinus* and *M. ganzhorni* within captive research colonies at the Duke Lemur Center and the Brunoy colony in France (Additional file 2: Table S8). Given this observation, it is possible that the genetic variation observed within captive research colonies of mouse lemurs historically recognized as ‘*M. murinus*’ is actually representative of either *M. murinus* or *M. ganzhorni* or both. We provisionally retain *M. murinus* as the appropriate taxonomic identification for the genome assembly presented herein until comparative genome sequence data and other relevant data are generated from wild individuals sampled from the type localities of both *M. murinus* and *M. ganzhorni* [45].

## Conclusions

The genus *Microcebus* constitutes a remarkable adaptive radiation of primates comprising at least 25 species distributed throughout and endemic to Madagascar [44]. The availability of a robust chromosome-level reference assembly, combined with novel biological insights into the mouse lemur centromere structure, creates new opportunities for analyses of evolutionary history, speciation mechanisms, and disease dynamics within *Microcebus*, and a greater general understanding of primate evolution. Moreover, the *M. murinus* genome will serve as an invaluable resource for a range of biomedical research areas. Comparisons of the content and function of the mouse lemur genome at both the nucleotide and structural level with that of other primates will allow researchers to reconstruct the content of the ancestral primate genome and, accordingly, provide insight for understanding the origin of primates. Genomic analyses among the strepsirrhine primates themselves will undoubtedly generate novel discoveries concerning this remarkable radiation that parallels the radiation of haplorrhine primates.

## Methods

### Individuals sequenced (Mmur 2.0)

Genomic DNA extracted from the samples listed in Additional file 2: Table S8 were used for Illumina and PacBio sequencing underlying the Mmur 2.0 genome assembly. All individuals are descendants of the historic captive colony originally established at the laboratory breeding colony of Brunoy (Muséum National d'Histoire Naturelle, UMR 7179 CNRS/MNHN, France; Agreement DDPP # D91-114-1). Recently, investigators have suggested recognition of two new species that were historically considered to be *M. murinus* [45], and this taxonomic revision would have possible implications for the nomenclature of the individuals used as sources of DNA for the work reported here. During our research, we identified the presence of mitochondrial haplotypes of both *M. murinus* and *M. ganzhorni* circulating within captive colonies (see Discussion and Additional file 2: Table S8). We defer final conclusions regarding taxonomic revision of the *M. murinus* complex and for the purposes of this genome assembly work use *M. murinus* as the relevant species designation. Further analysis of genetic, phenotypic, and behavioral diversity within *Microcebus* is clearly warranted before definitive conclusions concerning taxonomy can be drawn.

### Genome sequencing and primary assembly (Mmur 2.0)

We sequenced six Illumina libraries of nominal insert sizes 180 bp, 500 bp, 2 kb, 3 kb, 5 kb, and 8 kb for a total sequence coverage of approximately 190X (detailed methods for library construction is provided in Additional file 9: Supplementary Material). All raw sequences have been deposited at NCBI under BioProject PRJNA19967. Sequencing was performed on Illumina HiSeq 2000 instruments generating 100 bp PE reads. Reads were assembled using ALLPATHS-LG (v35218) [4] and further scaffolded and gap-filled using in-house tools Atlas-Link (v.1.0) and Atlas GapFill (v.2.2) (https://www.hgsc.bcm.edu/software/). Atlas-link is a scaffolding or super-scaffolding method that utilizes all unused mate pairs to increase scaffold sizes and create new scaffolds in draft-quality assemblies. Those modified scaffolds are then ordered and oriented. Atlas GapFill is run on a super-scaffolded assembly. Regions with gaps are identified and read mapping within or across those gaps are locally assembled using different assemblers (Phrap, Newbler and Velvet) in order to bridge the gaps with the most conservative assembly of previously unincorporated reads.

PBJelly (v14.9.9) [10] is a pipeline that improves the contiguity of draft assemblies by filling gaps, increasing contig sizes and super scaffolding by making use of long reads. We used 31.6X coverage of long Pacific Biosciences *RS I* and *RS II* sequences as input into PBJelly to improve the Atlas-Gapfill Illumina assemblies (PacBio sequence read length distributions provided in Additional file 10). For this step, we ran PBJelly in gap-fill + super-scaffold mode. The primary assembly (after PBJelly gap-filling of the Illumina-based ALLPATHS-LG assembly) was deposited at NCBI as Mmur 2.0 with a BioProject accession of PRJNA19967. We also ran PBJelly in gap-fill only mode on the assembly resulting from the second round Hi-C Lachesis analysis (see below).

### BioNano physical map production

High molecular weight DNA was extracted from leukocytes collected from a female mouse lemur born and housed at the Duke Lemur Center (DLC Animal ID: 7030). DNA extraction followed the BNG human blood DNA isolation protocol. White blood cells from approximately 400 μL of whole blood were washed after red blood cell lysis, embedded within agarose plugs, and digested with Protease-K. Purified DNA was labeled following the IrysPrep Reagent Kit protocol (BioNano Genomics). DNA was digested using the Nt.BspQI nicking endonuclease (New England Biolabs). Labeled DNA samples were loaded onto six IrysChips and run on the Irys imaging instrument. Over 1.5 million raw BNG physical map molecules were generated (minimum 100 kb, average of 9.62 labels per 100 kb, average length of 204.83 kb; Additional file 11: Figure S8) representing approximately 119X coverage of the mouse lemur genome. Consensus physical maps (CMAP) were assembled following established methods [46] with BNG molecules filtered at a minimum length of 150 kb and a minimum of eight labels (n = 986,806; ~89X coverage). A *P* value threshold for the BNG assembly was set to a minimum of 1 × 10^–10^ and molecule stretch was adjusted using AssembleIrysCluster.pl version 1.6.1 [46]. A total of 2915 CMAPs (N50: 1.108 Mb; total CMAP length: 2,322.056 Mb) were generated. CMAP and raw BNG molecules were deposited in NCBI under BioProject accession PRJNA19967.

### BioNano conflict resolution and hybrid scaffolding

BNG conflict resolution and hybrid-scaffolding steps used the IrysSolve 2.1 hybrid-scaffolding pipeline with input parameters following those optimized for human (details provided in Additional file 9: Supplementary Material; see BNG Hybrid Scaffolding Theory of Operation for a detailed explanation and summary of all input parameters; www.bionanogenomics.com). To summarize, the primary steps of BNG conflict resolution and hybrid scaffolding included the (1) creation of in silico physical maps for the input NGS genome assembly, (2) alignment of in silico NGS physical maps and BNG physical maps, and identification and resolution of conflicting alignments, (3) hybrid scaffold formation of non-conflicting NGS maps, (4) final alignment between NGS hybrid-scaffolds and BNG physical maps, and (5) FASTA file generation.

We performed two rounds of BNG conflict-resolution and hybrid scaffolding to identify and resolve putative scaffolding conflicts in both our primary Mmur 2.0 assembly and post Lachesis round 1 assembly (e.g., to resolve putative misassemblies introduced by Hi-C cross-chromosome 3D interactions). This iterative approach provided greater confidence in the long-range scaffolding of our final Mmur 3.0 assembly by providing an independent measure of accuracy through comparison of in silico maps with observed BNG physical maps, identification of specific genomic regions where in silico and BNG physical maps were in conflict, and resolution of those conflicts by breaking scaffolds in the NGS assembly. The information provided by these independent long-range datasets (BNG physical maps and Hi-C sequence data) were generated from the same *M. murinus* animal.

### Fibroblast cell line development and Hi-C

A 4-mm piece of dermal tissue was excised from the thigh of the same female mouse lemur used for BioNano physical maps (DLC 7030) and used for fibroblast isolation (see Additional file 9: Supplemental Material for detailed methodology). The in situ Hi-C library preparation was performed essentially as described by Rao et al. [47]. Two libraries were prepared and, for each library, 3 million fibroblast cells were crosslinked for 10 min with 1% formaldehyde. Nuclei were permeabilized and the DNA was digested with MboI restriction enzyme and ligated with T4 DNA ligase. The library was enriched for ligation products via biotinylation and prepared for sequencing on the Illumina platform. Prior to deep sequencing, approximately 1 million reads were sequenced from each library and processed with the Juicer pipeline [48] in order to perform quality control assessments, such as calculating the percent of read pairs representing Hi-C contacts as well as the frequency of the ligation motif.

A total of 2,094,030,784 Hi-C Illumina reads were generated. The reads were mapped to the first and second round BioNano scaffolds using BWA-MEM (v0.7.12) [49] with 98.96% of read mapping to either assembly. The Lachesis [23] PreprocessSAMs.pl script was used to remove reads not within 500 bp of a restriction site and remove pairs in which both reads were not mapped. This resulted in 1,658,366,836 remaining reads in the first round and 1,658,402,878 remaining reads in the second round. We ran Lachesis (v2151de9) using parameters based on the distributed test_case.ini file. The Lachesis pipeline uses three-dimensional chromatin-interaction information associated with Hi-C data to identify and arrange NGS scaffolds that putatively belong to individual chromosomes (reviewed in [23]).

### Assembly evaluation

The Benchmarking Universal Single-Copy Orthologs (BUSCO) tool (v1.1b1) [28] was used to assess the quality of gene models predicted on each of the mouse lemur assemblies. The vertebrate protein dataset consisting of 3023 proteins was used and the species was set to human, the only available primate, to use pre-computed Augustus metaparameters. Basic statistical descriptions of each assembly were generated using the assemblathon_stats.pl Perl script [34]. Statistics were calculated for both scaffold and contig sequences with contigs generated by splitting scaffolds on runs of 25 or more Ns. Mouse lemur canonical transcripts (16,319 protein coding and 8716 non-coding) were downloaded using the Ensembl API and mapped to each assembly using BLAT [50]. The total number of transcripts mapped at different percentages of aligned lengths was calculated. The Genome Analysis Toolkit (GATK; v3.3-0) [51], following GATK Best Practices [52], was used to call SNPs and indels based on Illumina BWA-MEM mappings of Illumina assembly sequences to the assemblies. Homozygous alternative SNP and indel calls were used as an estimate of assembly error rates. Pairwise alignments between the 33 primary mouse lemur Lachesis groups and 23 human chromosomes (hg38) were performed using MUMmer 3 [53], and resulting alignments were visualized using the Circos software package (v0.69) [54].

### Centromere characterization

#### Identification of centromere monomer

Raw C2 PacBio reads 8 kb and greater (25.19 Gb total sequence) were used for centromere monomer screening using TRF (v4.07) and the following parameters: match 2, mismatch 6, delta 6, PM 80, PI 10, minscore 50, and maxperiod 2000. The resulting TRF output was mined using custom scripts (https://github.com/cryancampbell/centromere_seeker) to extract all repeats having a minimum length of 10 bp, minimum tandem repeat unit of 4, and a minimum percent similarity of 70% (across the core monomer). Monomer length and overall repeat size were graphed using R to identify enriched monomers throughout the mouse lemur genome and the distinct signature of a commonly occurring 53 bp monomer (identified herein as Mm53) was observed (Fig. 5, see Results). This centromere discovery pipeline has since been automated to combine TRF, R, and the custom scripts; it is available at https://github.com/cryancampbell/centromere_seeker. This monomer sequence was extracted from corresponding PacBio reads using the TRF output and the Geneious software package (v8).

The genome-wide distribution of Mm53 was visualized using an Ultramer oligonucleotide probe and FISH, and confirmed to be associated with all mouse lemur centromeres except for the X chromosome (see below). After FISH confirmation, we utilized the new –l option in TRF v4.09 to identify arrays of Mm53 monomers within our final Mmur 3.0 assembly. Visualization of optical maps aligning to centromeric scaffolds was performed using IrysView software v2.5.

#### Cell culture and metaphase chromosome harvest

The primary mouse lemur fibroblasts were cultured in MEM alpha supplemented with 20% fetal bovine serum (FBS, Mediatech) and 1X antibiotic/antimycotic (Gibco). Low passage (p3–p5) cells were harvested for metaphase chromosome preparations by treating cells with 50 ng/mL nocodazole for 8–12 hours. Cells were isolated by trypsinization, and swollen in hypotonic solution (1:1:1 v/v/v 75 mM KCl/0.8% Na Citrate/dH_2_O) for 10 minutes at room temperature, before fixing 5–6 times in 3:1 methanol:acetic acid. Chromosome preparations were stored long-term at 4 °C.

#### FISH

An Ultramer oligonucleotide to the putative 53 bp centromeric sequence (Mm53; CGG-GCA-GGC-AGG-GCG-CAG-TGC-GGA-TCT-GGC-TGT-GTC-CAC-TCA-CCC-ACG-GCA-GA) containing 5’ biotin modification was synthesized by Integrated DNA Technologies, Inc. (Coralville, IA, USA). Mm53-bio (400 ng) was precipitated and resuspended in 15 μL of 50% hybridization mix (50% formamide, 20% dextran sulfate, 2X saline sodium citrate (SSC), 0.01% Triton X-100). Metaphase chromosomes that had been dropped onto clean glass slides were pre-treated with 0.05 mg/mL pepsin in 0.01 N HCl for 1 minute, followed by three washes in 2X SSC, and dehydration through an ice-cold ethanol series (70%, 95%, 100%). Slides were briefly air-dried and then incubated in 100 μg/mL RNase A/2X SSC at 37 °C for 30 minutes, and dehydrated in ethanol as before. Slides were denatured for 50 seconds in 70% formamide/2X SSC, pH 7.0 at 72 °C, and dehydrated in ethanol. The Mm53-bio probe was added to denatured slides, covered with a glass coverslip, and sealed with rubber cement. Hybridization was carried out in a humidified chamber overnight at 37 °C. Following hybridization, slides were washed four times in 50% formamide/2X SSC/0.05% Tween-20 (SSCT) for 5 minutes each, followed by four washes in 2X SSCT for 2 minutes each. Slides were incubated in 4X SSCT for 5 minutes, blocked in 5% nonfat milk diluted in 4X SSC for 10 minutes at room temperature, and incubated with Alexa Fluor 488-streptavidin (Invitrogen) for 1 hour at room temperature. After three washes in 4X SSCT, slides were counterstained with 1 mg/mL DAPI diluted in Vectashield (V-DAPI; Vector Laboratories) and covered with a glass coverslip.

#### Combined immunofluorescence and FISH (IF-FISH)

We used an adaptation of our standard protocol [55] to obtain three-dimensionally preserved metaphase chromosomes from mouse lemur cells. Low passage fibroblast cultures were incubated with 100 ng/mL nocodazole for 3 hours at 37 °C, and mitotic cells were collected by shake-off. Cells were diluted to 4 × 10^4^ cells/mL in 1:1:1 hypotonic (see above) and incubated at room temperature for 10 minutes, before loading 500 μL of cell solution into single chamber cytofunnels. Cells were centrifuged in a Shandon Cytospin 4 at 2000 rpm for 5 minutes, followed by a 5 minute incubation in KCM (10 mM Tris pH 8.0; 120 mM KCl; 20 mM NaCl; 0.5 mM EDTA; 0.1% Triton X-100), and fixation for 10 minutes at room temperature in 2% paraformaldehyde in 1X PBS. Cells were blocked (1X PBS, 5% BSA, 0.5% Triton X-100) for 30 minutes at room temperature, before the addition of human CENP-A antibodies (custom polyclonal CENP-A, 1:300 [56]), and incubated overnight at 4 °C. Following three washes in room temperature KCM, slides were incubated with secondary antibodies (Alexa Fluor donkey anti-rabbit; Invitrogen) for 2 hours at room temperature. Slides were washed as before, then antibody-protein complexes were crosslinked using 10% formalin. Slides were stored in KCM until FISH, which was performed essentially as described above, except pepsin and RNase treatments were omitted, and slides were denatured in 70% formamaide/2X SSC, pH 7.0 at 74 °C for 5 minutes before application of Mm53-bio probe and hybridization overnight at 37 °C.

#### Microscopy

All images were acquired using an inverted Olympus IX-71 microscope connected to the Deltavision Elite imaging system (Applied Precision/GE Healthcare) equipped with a Photometrics CoolSNAP HQ^2^ CCD camera and running the SoftWoRx imaging software. IF-FISH images were captured using a 100X objective (NA 1.40) collected as z-stacked images (0.1 mm between sections) that were binned at 2 × 2. Images were quick projected, collapsing *z*-stacks into a single image that was saved as a PSD file and exported to Adobe Photoshop. Coincidence of Mm53 and CENP-A was analyzed using the JACoP plugin in Image J, as well as RGB profile line plots.

## Additional files

Additional file 1: Figure S1. Detailed flowchart of methods used herein for the de novo assembly of the gray mouse lemur (*Microcebus murinus*). The initial assembly was generated using Illumina data and AllPaths-LG, followed by refined scaffolding using Atlas-Link and gap filling using Atlas-GapFill. Further gap filling with PacBio data and PBJelly followed generating Mmur 2.0. The Mmur 2.0 assembly was super-scaffolded in an iterative method using BNG optical map data to identify conflicts, break scaffolds and join other scaffolds, and identify Lachesis and Hi-C proximity ligation data to further super-scaffold. The PBJelly method was used a second time to fill gaps in the final super-scaffolds, creating the Mmur 3.0 assembly. Asterisks indicate PBJelly2 and Pilon used the PacBio and Illumina datasets at the top of the diagram, respectively. (PDF 1157 kb)

Additional file 2: Table S1. Quality assessment and assembly statistics of iterative genome assemblies of *Microcebus murinus.* **Table S2.** BioNano Genomics in silico physical map production, conflict resolution, and hybrid scaffolding statistics for two *M. murinus* assemblies (see Fig. 1). **Table S3.** Hi-C mapping and Lachesis assembly statistics. Statistics for mapping of Hi-C Illumina reads to BioNano generated assemblies are shown. Mapping percentages are based on a total of 2,094,030,784 sequenced Hi-C Illumina reads. Lachesis generated clustering, ordering, and orienting assembly statistics based on the Hi-C mappings for the two rounds of Lachesis are also shown. **Table S4.** Pilon error correction. The number of bases and indels corrected by Pilon after the application of PBJelly are shown. The length of corrected indel bases are also shown. **Table S5.** GATK estimates of SNP and indel error rates. Homozygous alternative (non-reference) alleles provide estimates of base and indel error rates in the assembly. A caveat of this is that assembled Illumina reads and PacBio reads are from different individuals. **Table S6.** Comparative cytogenetic data showing homologous chromosomes between human and mouse lemur. Data summarized from [29], with addition of the X chromosome (*inter* intercalated, *min* minute, *prox* proximal, *ter* terminal). **Table S7.** Mouse lemur chromosome assignments to the 33 Lachesis groups identified herein (see Fig. 4 and Additional file 4: Figure S3). Bold font identifies the 23 chromosomes that are supported by comparative cytogenetic data ([29]; Fig. 4, Additional file 2: Table S6). The remaining 10 chromosomes are putative assignments pending FISH confirmation. **Table S8.** *Microcebus murinus* samples used for genetic data presented herein. Mitochondrial haplotype identification for each sample is based on phylogenetic analyses of 1140 bp of the cytochrome-b gene. *BCM* Baylor College of Medicine, *DLC* Duke Lemur Center. (XLSX 36 kb)

Additional file 3: Figure S2. Sequence length distribution of regions between BNG conflicts of final Mmur 3.0 assembly. Sequences (contigs) are arranged from longest to smallest along the X-axis. The L50 statistic shows that 50% of the genome is contained in 47 contigs and the L75 statistic shows 75% of the genome is contained in 102 contigs (separated by BNG cut sites). (PDF 1112 kb)

Additional file 4: Figure S3. Circos diagram showing major regions of synteny between the 33 mouse lemur Lachesis scaffolds and human chromosomes (see Fig. 4). The legend identifies mouse lemur chromosomes that align with human chromosomes in patterns that are consistent with previously published comparative cytology results (see Results). (JPG 563 kb)

Additional file 5: Figure S4. Example of tandemly repeated Mm53 monomer identified in the Mmur 3.0 genome assembly (~26 of ~144 monomers shown from Super-Scaffold_6125). A FISH probe binding to this monomer localized to the majority of mouse lemur centromeres (see Figs. 2 and 6). (PDF 104 kb)

Additional file 6: Figure S5 A–D. Consensus BioNano physical maps (blue) aligning to and extending beyond mouse lemur genome scaffolds (green) that terminate in the Mm53 monomer. A BNG label site (repeat unit ~3.9 kb) is shown within mouse lemur centromeric regions (black arrows). **E**. Scaffold (green) aligned to BNG physical map (blue). An N gap of approximately 500 kb is shown in the center of the scaffold; however, optical map shows a putative centromere at the same location. **F**. Magnified region of the repetitive BNG label that identifies putative higher-order repeat structure. Each label (or nick-site) is separated by approximately 3.9 kb. (PDF 247 kb)

Additional file 7: Figure S6. Repeat unit size (in kilobases; X-axis) versus number of repeat units per raw BioNano physical map (Y-axis) (see Results). Blue line indicates common repeat unit of approximately 2.6 kb detected in the mouse lemur genome (with a tandem repeat signature at ~5.2 kb (second blue line)). Red line shows approximately 3.9 kb repeat unit and this repeat length is consistent with putative higher order repeat length detected in mouse lemur centromeres (second red line shows tandem repeat at ~7.8 kb). (PDF 120 kb)

Additional file 8: Figure S7 A–D. Representative (4 of 29) BioNano physical maps showing putative mouse lemur centromeres. Putative higher order repeat unit within each array is ~3.9 kb (see Figs. 5 and 6, Additional file 6: Figure S5). (PDF 427 kb)

Additional file 9: Supplementary materials. (DOCX 22 kb)

Additional file 10: Read length statistics and graphs for PacBio RS I and RS II sequence data (see tabs below). (XLSX 55 kb)

Additional file 11: Figure S8. BNG physical map molecule size distribution (n = 1,573,503) for raw *Microcebus murinus* physical maps (see Methods). (PDF 47 kb)
