# Supplementary figures and images for: Hybrid de novo genome assembly and centromere characterization of the gray mouse lemur (Microcebus murinus)
Source: BMC Biol. 2017 Nov 16;15:110. doi: 10.1186/s12915-017-0439-6 (PMC5689209; doi:10.1186/s12915-017-0439-6)

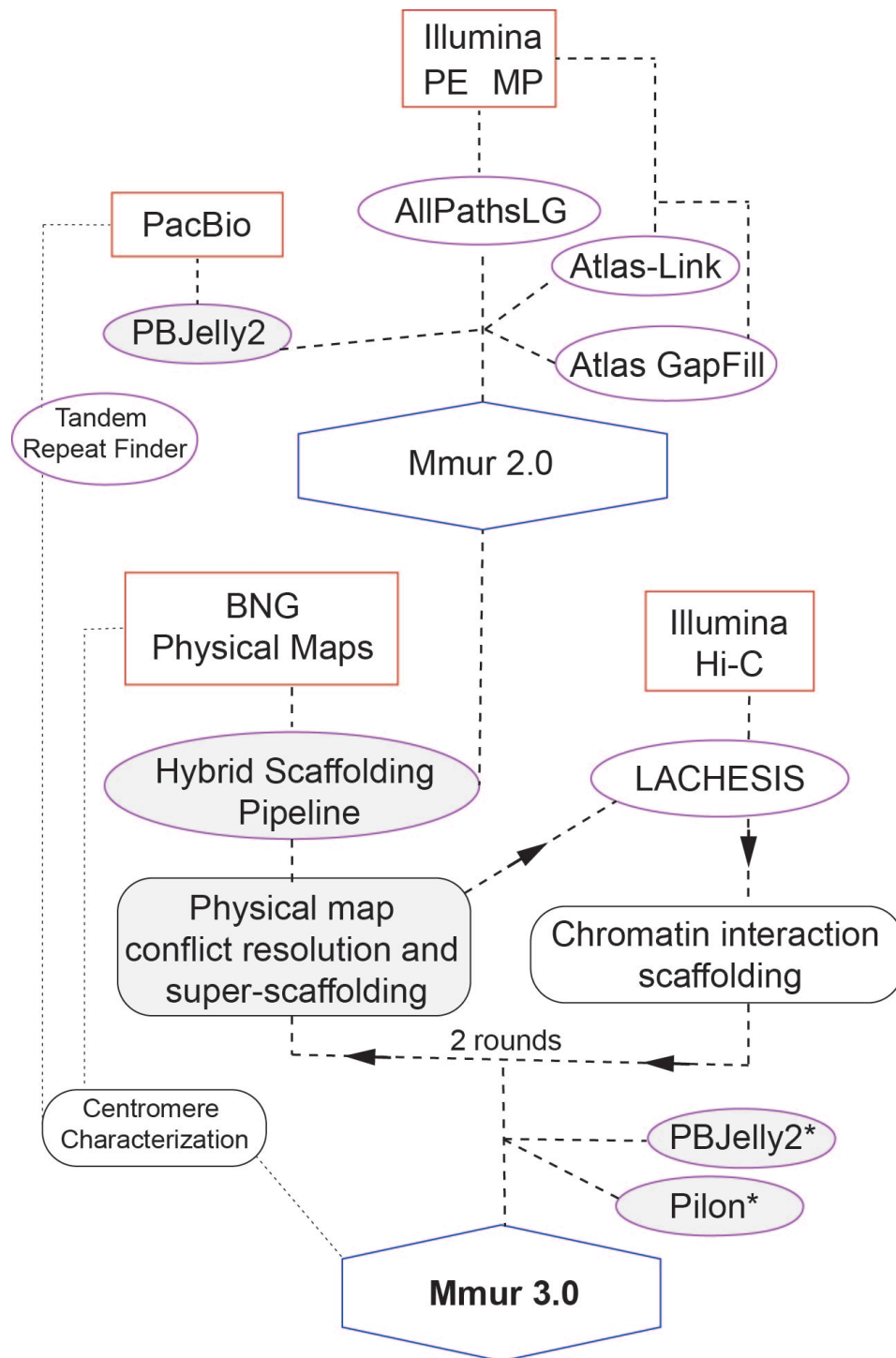

Supplement: Supplementary file 1 — Detailed flowchart of methods used herein for the de novo assembly of the gray mouse lemur (Microcebus murinus). The initial assembly was generated using Illumina data and AllPaths-LG, followed by refined scaffolding using Atlas-Link and gap filling using Atlas-GapFill. Further gap filling with PacBio data and PBJelly followed generating Mmur 2.0. The Mmur 2.0 assembly was super-scaffolded in an iterative method using BNG optical map data to identify conflicts, break scaffolds and join other scaffolds, and identify Lachesis and Hi-C proximity ligation data to further super-scaffold. The PBJelly method was used a second time to fill gaps in the final super-scaffolds, creating the Mmur 3.0 assembly. Asterisks indicate PBJelly2 and Pilon used the PacBio and Illumina datasets at the top of the diagram, respectively. (PDF 1157 kb) [file 12915_2017_439_MOESM1_ESM.pdf]

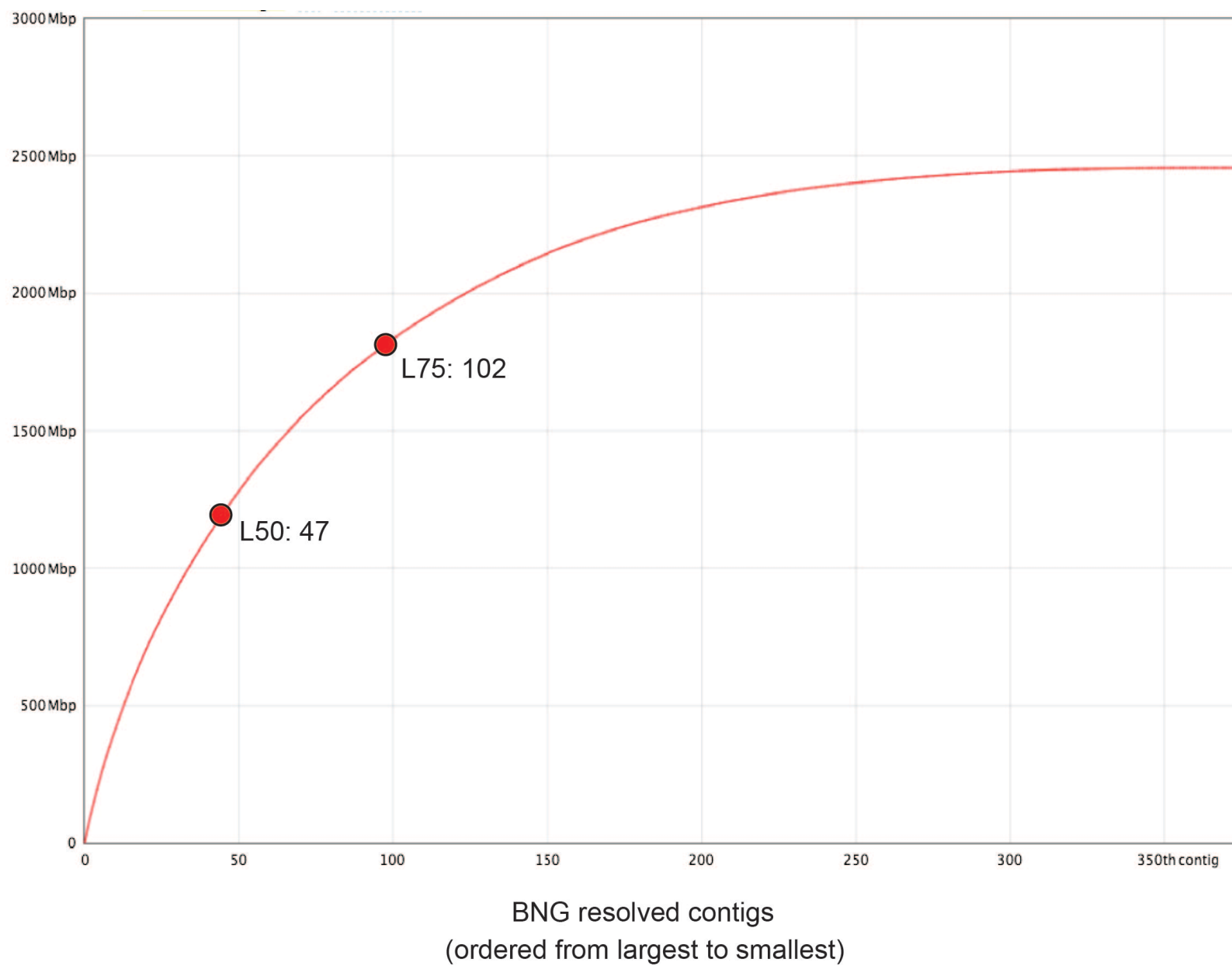

Supplement: Supplementary file 3 — Sequence length distribution of regions between BNG conflicts of final Mmur 3.0 assembly. Sequences (contigs) are arranged from longest to smallest along the X-axis. The L50 statistic shows that 50% of the genome is contained in 47 contigs and the L75 statistic shows 75% of the genome is contained in 102 contigs (separated by BNG cut sites). (PDF 1112 kb) [file 12915_2017_439_MOESM3_ESM.pdf]

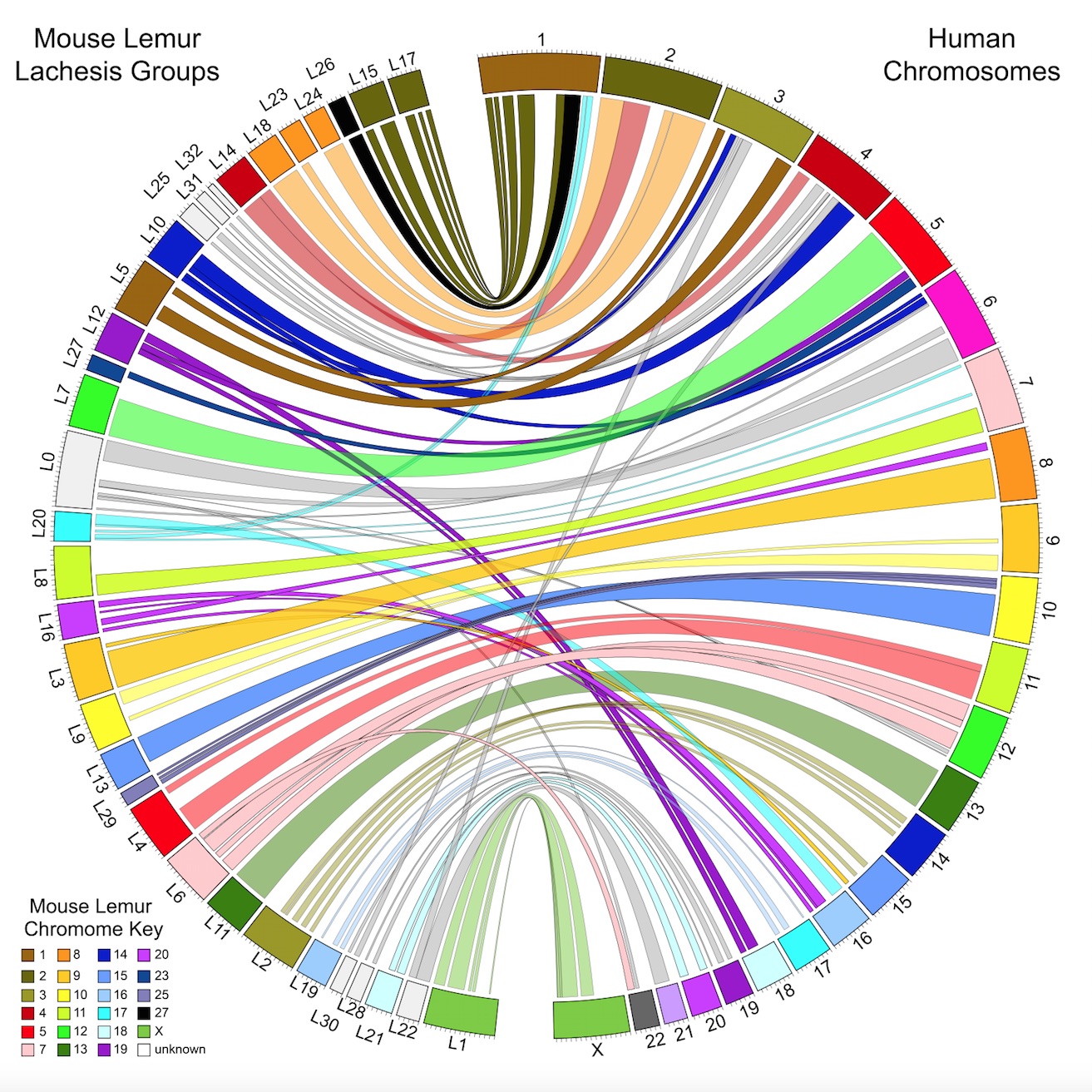

Supplement: Supplementary file 4 — Circos diagram showing major regions of synteny between the 33 mouse lemur Lachesis scaffolds and human chromosomes (see Fig. 4). The legend identifies mouse lemur chromosomes that align with human chromosomes in patterns that are consistent with previously published comparative cytology results (see Results). (JPG 563 kb) [file 12915_2017_439_MOESM4_ESM.jpg]

A.

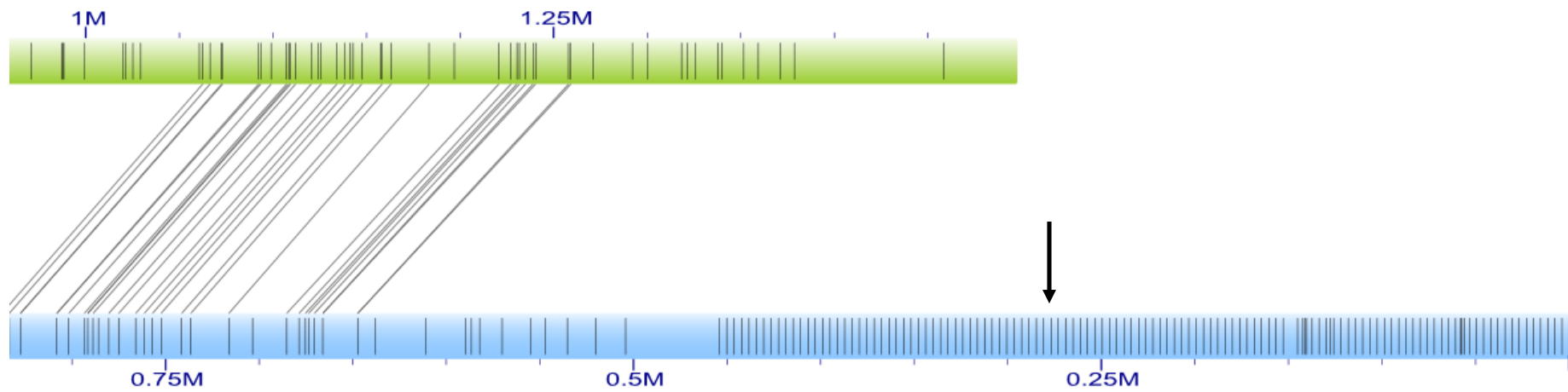

B.

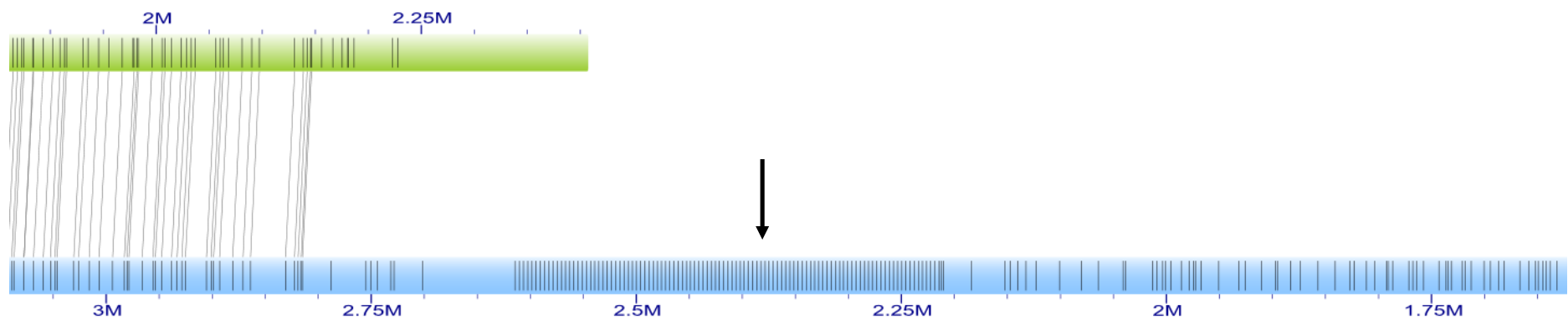

C.

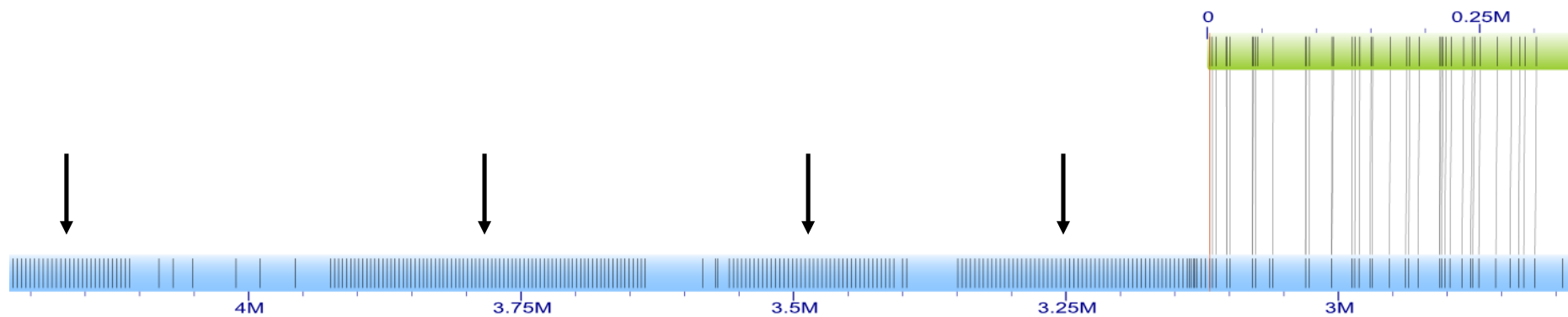

D.

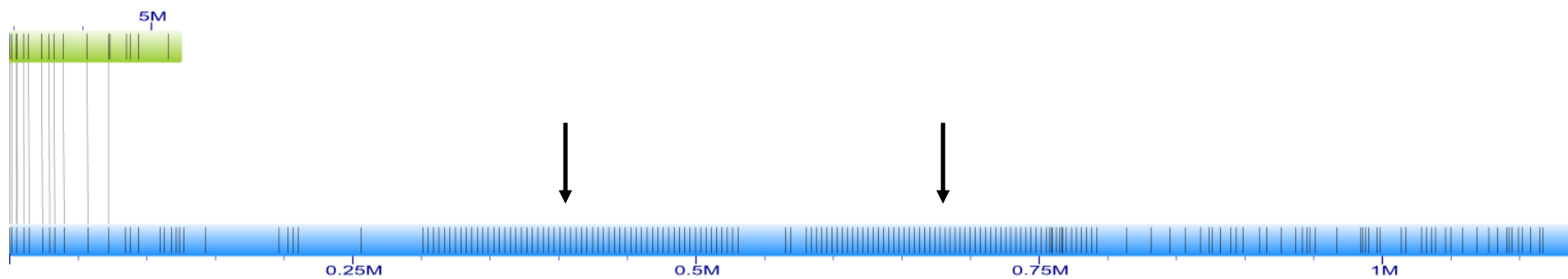

E.

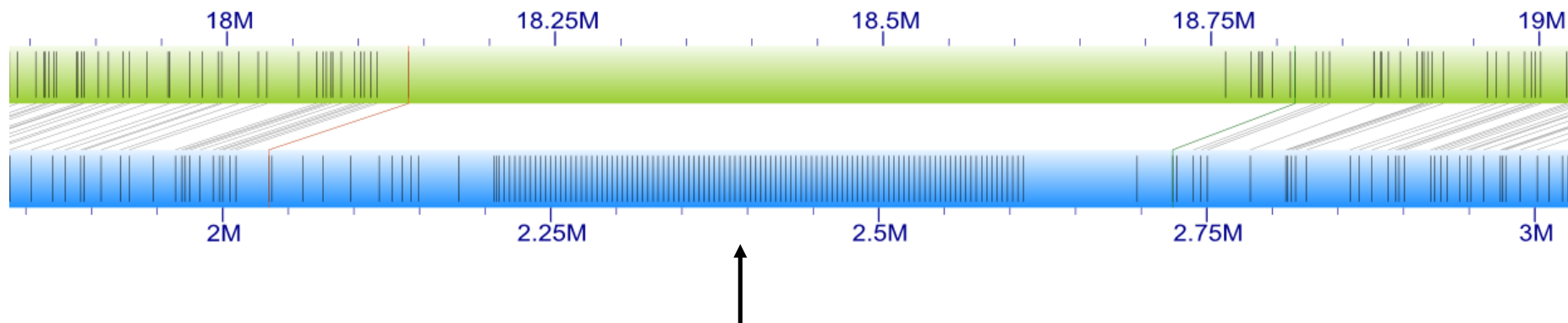

F.

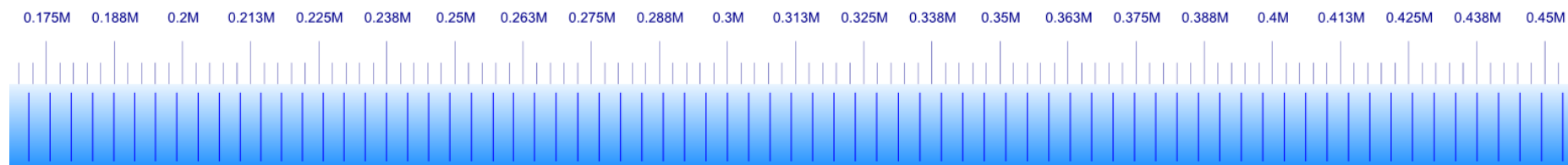

Supplement: Supplementary file 6 — Consensus BioNano physical maps (blue) aligning to and extending beyond mouse lemur genome scaffolds (green) that terminate in the Mm53 monomer. A BNG label site (repeat unit ~3.9 kb) is shown within mouse lemur centromeric regions (black arrows). E. Scaffold (green) aligned to BNG physical map (blue). An N gap of approximately 500 kb is shown in the center of the scaffold; however, optical map shows a putative centromere at the same location. F. Magnified region of the repetitive BNG label that identifies putative higher-order repeat structure. Each label (or nick-site) is separated by approximately 3.9 kb. (PDF 247 kb) [file 12915_2017_439_MOESM6_ESM.pdf]

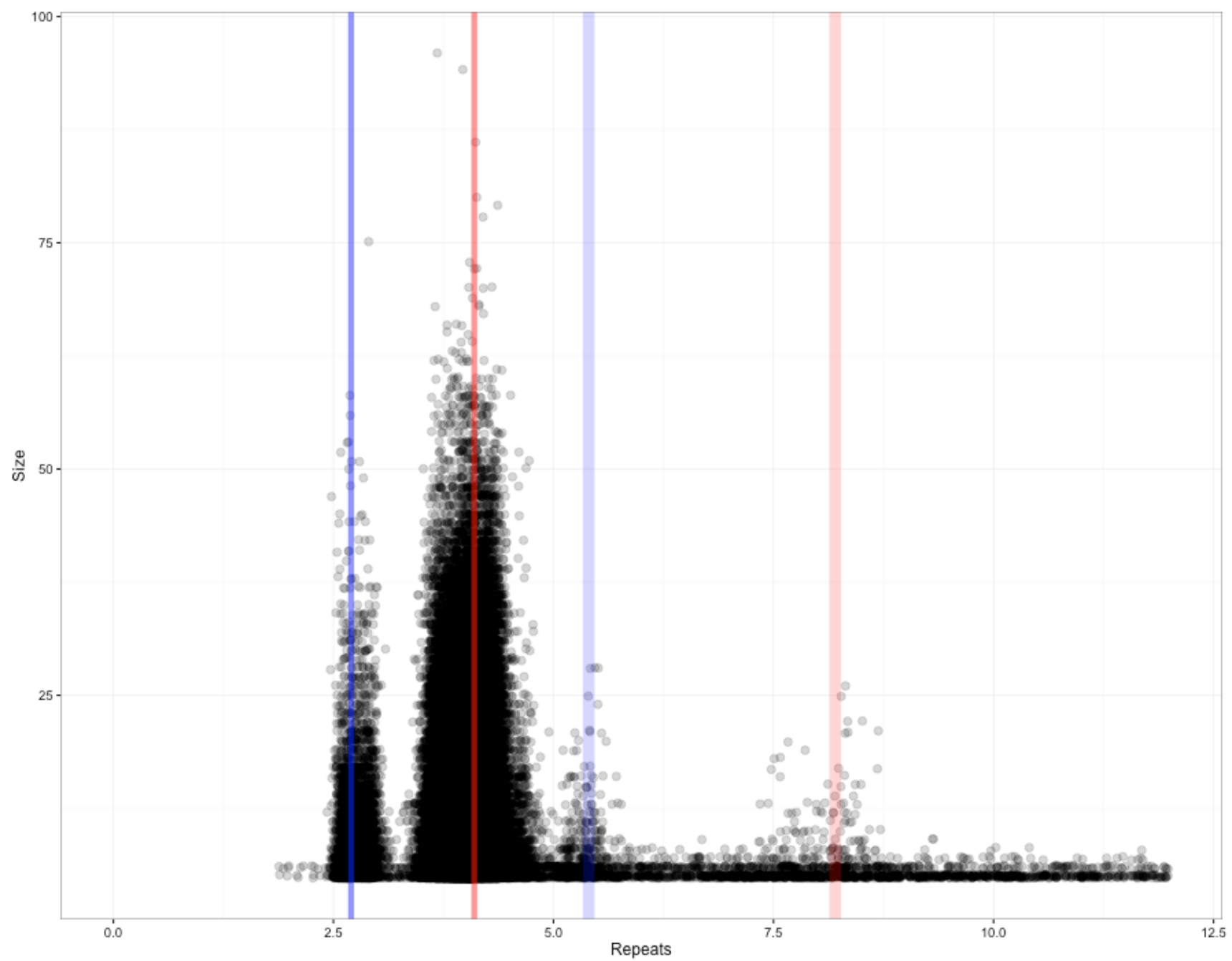

Supplement: Supplementary file 7 — Repeat unit size (in kilobases; X-axis) versus number of repeat units per raw BioNano physical map (Y-axis) (see Results). Blue line indicates common repeat unit of approximately 2.6 kb detected in the mouse lemur genome (with a tandem repeat signature at ~5.2 kb (second blue line)). Red line shows approximately 3.9 kb repeat unit and this repeat length is consistent with putative higher order repeat length detected in mouse lemur centromeres (second red line shows tandem repeat at ~7.8 kb). (PDF 120 kb) [file 12915_2017_439_MOESM7_ESM.pdf]

A.

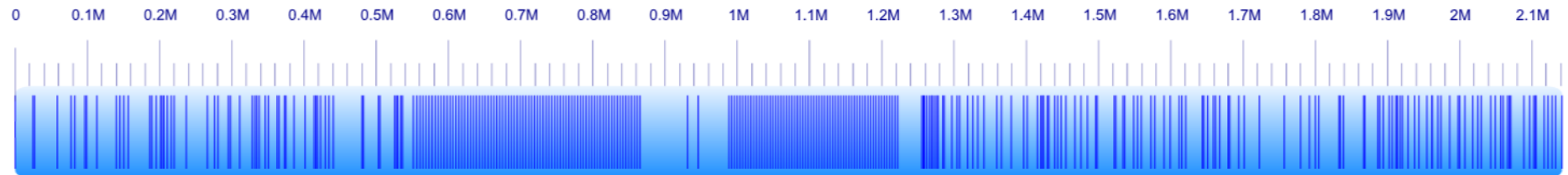

B.

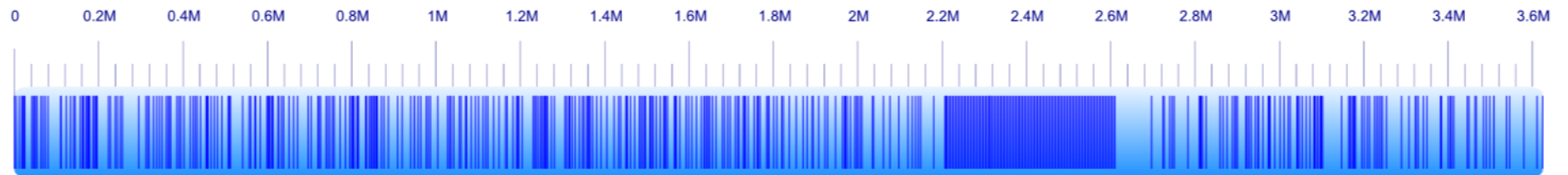

C.

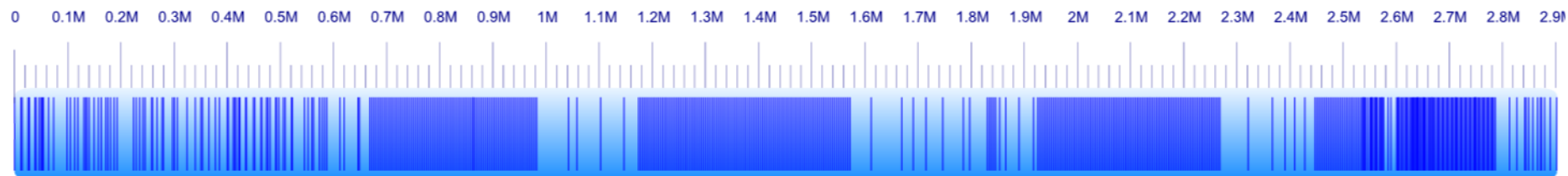

D.

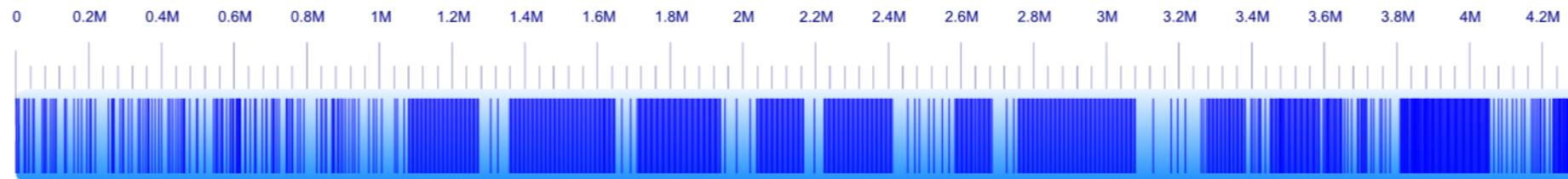

Supplement: Supplementary file 8 — Representative (4 of 29) BioNano physical maps showing putative mouse lemur centromeres. Putative higher order repeat unit within each array is ~3.9 kb (see Figs. 5 and 6, Additional file 6: Figure S5). (PDF 427 kb) [file 12915_2017_439_MOESM8_ESM.pdf]

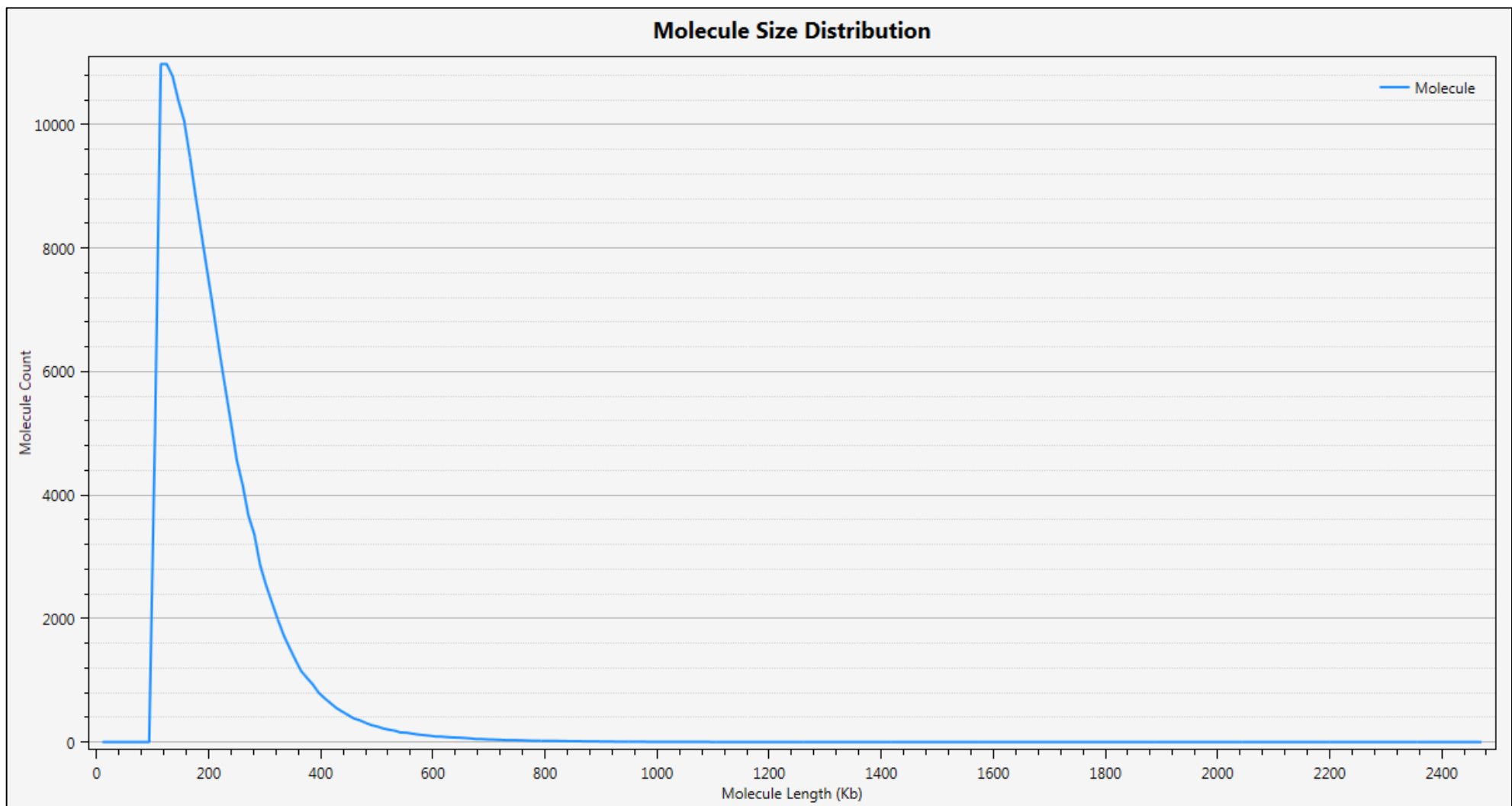

Supplement: Supplementary file 11 — BNG physical map molecule size distribution (n = 1,573,503) for raw Microcebus murinus physical maps (see Methods). (PDF 47 kb) [file 12915_2017_439_MOESM11_ESM.pdf]
