# Supplementary material for: Hybrid de novo genome assembly and centromere characterization of the gray mouse lemur (Microcebus murinus)
Source: BMC Biol. 2017 Nov 16;15:110. doi: 10.1186/s12915-017-0439-6 (PMC5689209; doi:10.1186/s12915-017-0439-6)
Supplement: Supplementary file 5 — Example of tandemly repeated Mm53 monomer identified in the Mmur 3.0 genome assembly (~26 of ~144 monomers shown from Super-Scaffold_6125). A FISH probe binding to this monomer localized to the majority of mouse lemur centromeres (see Figs. 2 and 6). (PDF 104 kb) [file 12915_2017_439_MOESM5_ESM.pdf]

```
4,860      14,870     14,880     14,890     14,900     14,910     14,920     14,930     14,940     14,950     14,960     14,970     14,980
CCCGTCTGCCGTGGGTGTGTGGACATAGCCAGATCCGCACTGTGCCTTGCCTGCCCGTCTGCCGTGGGCGAGTGGACACAGCCAGATCCGCACTGCGCCCTTCCTGCCCGTCTCCCGTGGGCGAGTG

  14,990     15,000     15,010     15,020     15,030     15,040     15,050     15,060     15,070     15,080     15,090     15,100     15,110
GACACAGCCAGATCCGCACTGCGCCCTGCCTGCCCGTCTGCCGTGGGCGAGTGGACACAGCCAGATCCGCACTGCGCCCTGCCTGTCCGTCTGCCGTGGGGACTGGACACAGCCAGATCCGCACTG

     15,120     15,130     15,140     15,150     15,160     15,170     15,180     15,190     15,200     15,210     15,220     15,230     15,24
CGCCCTGCCTGCACGTCTCCCGTGGGTTAGTGGACACAGCCAGATCCGCACTGCGCCCTGCCTGCCCGTCTGCCGTGGGTGAGTGGACACAGCCAGATCCGCACTGTGCCCTGCCTGCCCGTCTGCC

         15,250     15,260     15,270     15,280     15,290     15,300     15,310     15,320     15,330     15,340     15,350     15,360
CTGGGTGAGGGGACACAGCCAGATCCGCACTGCGCCCTGCCTGCCCGTCTCCCGTGGGTGAGTGGACACAGCCAGATCTGCACTGCGCCCTGCCTGCCCGTCTGCCGTGGGTTAGTGGACATAGCCA

 15,370     15,380     15,390     15,400     15,410     15,420     15,430     15,440     15,450     15,460     15,470     15,480     15,490
TATCCACACAGCACCCTCTCTGCCCGTCTCCCGTGGGTGAGTGGACACAGCCAGATCCGCACTGCGCCTTGCCTGCCCGTCTGCCGTGGGTCAGTGGACATAGCCAGATCCACACAGCACCGTCTCT

    15,500     15,510     15,520     15,530     15,540     15,550     15,560     15,570     15,580     15,590     15,600     15,610     15,620
GCCCGTCTCCCGTGGGTGAGTGGACACAGCCAGATCCGCACTGCGCCTTGCCTGCCCGTCTCCCGTGGGTGAGTGGACACAGCCAGATCCGCACTGCGCCCTGCATGCCCATCTCCCGTGGGTGAGT

        15,630     15,640     15,650     15,660     15,670     15,680     15,690     15,700     15,710     15,720     15,730     15,740
GGACACAGCCAGATCCGCACTGCGCCTTCCCTGCCCGTCTCCCGTTGGCGAGTGGACACAGCCAGATCCGCACTGCGCCCTGCCTGCCCGTCTCCCGTGGGTGAGTGGACACAGCCAGATCCACACT

15,750     15,760     15,770     15,780     15,790     15,800     15,810     15,820     15,830     15,840     15,850     15,860     15,870
GCGCCCTGCCTGCCGGTCTCCCGTGGGTGAGTGGACACAGCCAGATCCGCACTGCGCCCTGCCTGCCCGTCTGCCGTGGGCGAGTGGACACAGCCAGATCCGCACTGCGCGCTGCCTGCCCGTCTCC

   15,880     15,890     15,900     15,910     15,920     15,930     15,940     15,950     15,960     15,970     15,980     15,990     16,000
CGTGGGCGAGTGGACACAGCCAGATCCGCACTGCGCCCTGCCTGCCCGTCTGTCGTGGGCGAGTGGACACAGCCAGATCCGCACTGCACCCTGCCTGACCGTCTGCCGTCGGTGAGTGGACACAGCC

       16,010     16,020     16,030     16,040     16,050     16,060     16,070     16,080     16,090     16,100     16,110     16,120
AGATGACACAGCCAGATCCGCACTGCGCGCTGCCTGCCCGTCTGTCGTGGGCGAGTGGACACAGCCATATCCGCACTGCGCCCTGCCTGCCCGTCTGCCGTGGGTGAGTGGACACAGCCAGATCCAC

6,130      16,140     16,150     16,160     16,170     16,180     16,190     16,200     16,210     16,220     16,230     16,240     16,250
ACTGCGCCCTGCCTGCCCGTCTGCCGTGGGTCAGTGGACATAGCCAGATCCACACAGCGCCCTTTCTGCCCGTCTCCCGTGGGTGAGTGGACACAGCCAGATCCACACGGCGCCCTGCCTGCCCTTT
```
