# Supplementary material for: Hybrid de novo genome assembly and centromere characterization of the gray mouse lemur (Microcebus murinus)
Source: BMC Biol. 2017 Nov 16;15:110. doi: 10.1186/s12915-017-0439-6 (PMC5689209; doi:10.1186/s12915-017-0439-6)
Supplement: Supplementary file 9 — Supplementary materials. (DOCX 22 kb) [file 12915_2017_439_MOESM9_ESM.docx]

**Supplemental Material**

**Larsen and Harris et al.** Hybrid *de novo* genome assembly and centromere characterization of the gray mouse lemur (*Microcebus murinus*).

**Supplemental Methods**

***Illumina library preparation***

Six Illumina libraries of nominal insert sizes 180 bp, 500 bp, 2 kb, 3 kb, 5 kb and 8 kb were constructed. To prepare the 180 bp and 500 bp libraries, we used a gel-cut paired end (PE) library protocol. Briefly, 1 µg of the DNA was sheared using a Covaris S-2 system (Covaris, Inc. Woburn, MA) using the 180 bp or 500 bp program. Sheared DNA fragments were purified with Agencourt AMPure XP beads, end-repaired, dA-tailed, and ligated to Illumina universal adapters. After adapter ligation, DNA fragments were further size selected by agarose gel and PCR amplified for 6 to 8 cycles using Illumina P1 and Index primer pair and Phusion® High-Fidelity PCR Master Mix (New England Biolabs). The final library was purified using Agencourt AMPure XP beads and quality assessed by Agilent Bioanalyzer 2100 (DNA 7500 kit) to determine library quantity and fragment size distribution before sequencing.

Long mate pair (MP) libraries with 2 kb, 3 kb, 5 kb and 8 kb insert sizes were constructed according to the manufacturer’s protocol (Mate Pair Library v2 Sample Preparation Guide art # 15001464 Rev. A PILOT RELEASE). Briefly, 5 µg (for 2 and 3 kb size libraries) or 10 µg (5 and 8 kb libraries) of genomic DNA was sheared to desired size by Hydroshear (Digilab, Marlborough, MA), then end repaired and biotinylated. Fragment sizes between 1.8–2.5 kb (2 kb) 3.0–3.7 kb (3 kb), 4.5–6.0 kb (5 kb) or 8–10 kb (8 kb) were purified from 1% low melting agarose gel and circularized by blunt-end ligation. These size selected circular DNA fragments were then sheared to 400 bp (Covaris S-2), purified using Dynabeads M-280 Streptavidin Magnetic Beads, end-repaired, dA-tailed, and ligated to Illumina PE sequencing adapters. DNA fragments with adapter molecules on both ends were amplified for 12 to 15 cycles with Illumina P1 and Index primers. Amplified DNA fragments were purified with Agencourt AMPure XP beads. Quantification and size distribution of the final library was determined as described above prior to sequencing.

***BioNano conflict resolution and hybrid scaffolding parameters***

We implemented the conservative BNG hybrid scaffolding approach recommended for genomes approximately 3 Gb in size, with initial alignment and conflict detection P-values being 1.0E-10 and 1.0E-13, respectively. Hybrid scaffolding pair merge alignment length was set a 160 kb with a merge P-Value of 1.0E-13 and the final alignment P-Value was 1.0E-10. *In silico* FASTA to CMAP conversion was performed using IrysSolve 2.1 and the recognition site for the BspQI restriction enzyme, and employed a minimum length threshold of 20 kb with 5 labels per molecule. The chimeric quality scores (i.e., the percent of BNG physical maps aligning fully to the left and right of each *in silico* NGS label) used to identify genomic regions in which NGS scaffolds and BNG physical maps were in conflict were generated using the *-y* flag and filtered BNG molecules. In brief, the BNG pipeline examines physical map coverage and calculates the chimeric quality scores to localize genomic segments exhibiting scaffold map conflicts. When the chimeric quality score exceeded 35%, with a minimum of 10 aligning BNG physical maps and window size of 10kb, we broke the NGS assembly at that location. We performed this BNG physical map conflict resolution using the hybrid-scaffolding pipeline on both the ALLPATHS-LG Mmur 2.0 assembly and the Lachesis round 1 assembly (Figure 1; Supplemental Figure S1). A final BNG analysis was performed on the Mmur 3.0 assembly to evaluate long-range scaffolding patterns introduced in the final Lachesis assembly.

***Fibroblast cell line development***

The 4mm biopsied dermal tissue was incubated in 70% EtOH for 5 minutes followed by 3 washes in sterile 1X PBS and then complete growth media (DMEM containing 4.5 g/L glucose, 110 mg/L sodium pyruvate supplemented with 1X antibiotic/antimycotic and 20% fetal bovine serum – not heat inactivated). Tissue pieces were incubated in a 1X final concentration (100 U/mL) of collagenase type II (Gibco 17101-015) diluted in calcium and magnesium-containing HBSS for 6 hours in a 37°C tissue culture incubator (5% CO2). Digested material was strained through a 100 µM cell strainer and centrifuged at 1,000 rpm for 5 minutes at room temperature. The HBSS was aspirated, and the remaining pellet resuspended and plated into one well of a 24-well plate using 1 mL of complete growth media. All unstrained material was placed back into fresh 1X collagenase type II diluted in complete media and incubated overnight in a 37°C tissue culture incubator. The digested tissue was again strained, centrifuged and plated into a separate well of a 24-well plate using 1 mL of complete growth media. Cells were designated as passage 0, and standard methods were used for cell expansion.

**Supplemental Text**

***Genome assembly cost estimates***

It is difficult to accurately estimate the cost associated with our assembly of the *Microcebus murinus* genome. This is because genomic technologies are experiencing rapid development (both hardware and sequencing chemistries) and consumer prices are in flux, thus costs can vary widely from institution to institution. Sequence lengths and throughput for Illumina, PacBio (Sequel), and BioNano have increased substantially over the past year. This observation, combined with improvements of genome assembly algorithms, contributes to the production of cost-effective chromosome-scale assemblies using hybrid methodologies similar to ours. Advertised prices do not include all costs (machine amortization, institutional overheads, academic discounts) and the added cost from failures of difficult protocols. We generated sequence and physical mapping data from 2011-2015, and the prices provided here reflect the technologies available during that timeframe. We provide the following estimates to help establish a record so that future comparisons can be made with respect to mammalian chromosome-level genome assemblies.

Ballpark estimates without overhead are ~$28,500 for a comparable Illumina HiSeq 2000 (~190x coverage) AllPathsLG data set; ~$38,300 for 20x PacBio *RS* *II* data set; ~$8,000 for a comparable 119X (raw molecules) BioNano Genomics data set (filtered consensus physical maps at ~89x); and ~$6,600 for a 70x Illumina Hi-C data set. The total estimated cost for libraries and sequencing associated with the gray mouse lemur genome would be approximately $81,400. The computational resources required to make use of these data are also not trivial and compute and storage charges are institution dependent. Moreover, advanced computational resources, such as the Extreme Science and Engineering Discovery Environment (XSEDE), are available to researchers. With Amazon Cloud Computing and storage prices (as of December 2016), we estimate an additional $76,510 would be needed to perform the analyses presented herein. For comparison, a *de novo* PacBio *RS II* ~60x data would be $114,900, with an additional $270,00 to $430,000 for the Amazon compute and storage. Computational requirements for all software packages used here are provided by each software’s developer (website or citation).
